# Local admixture of amplified and diversified secreted pathogenesis determinants shapes mosaic *Toxoplasma gondii* genomes

**DOI:** 10.1038/ncomms10147

**Published:** 2016-01-07

**Authors:** Hernan Lorenzi, Asis Khan, Michael S. Behnke, Sivaranjani Namasivayam, Lakshmipuram S. Swapna, Michalis Hadjithomas, Svetlana Karamycheva, Deborah Pinney, Brian P. Brunk, James W. Ajioka, Daniel Ajzenberg, John C. Boothroyd, Jon P. Boyle, Marie L. Dardé, Maria A. Diaz-Miranda, Jitender P. Dubey, Heather M. Fritz, Solange M. Gennari, Brian D. Gregory, Kami Kim, Jeroen P. J. Saeij, Chunlei Su, Michael W. White, Xing-Quan Zhu, Daniel K. Howe, Benjamin M. Rosenthal, Michael E. Grigg, John Parkinson, Liang Liu, Jessica C. Kissinger, David S. Roos, L David Sibley

**Affiliations:** 1 Department of Infectious Diseases, The J. Craig Venter Institute, 9704 Medical Center Drive, Rockville, Maryland 20850, USA; 2 Department of Molecular Microbiology, Washington University School of Medicine, 660 S. Euclid Avenue, St Louis, Missouri 63130, USA; 3 Laboratory of Parasitic Diseases, NIAID, National Institutes of Health, Bethesda, Maryland 20892, USA; 4 Pathobiological Sciences, School of Veterinary Medicine, Louisiana State University, Baton Rougea Louisian 70803, USA; 5 Department of Genetics, University of Georgia, Athens, Georgia 30602, USA; 6 Center for Tropical and Emerging Global Diseases, University of Georgia, Athens Georgia 30602, USA; 7 Program in Molecular Structure and Function, Hospital for Sick Children, Toronto, Ontario, Canada M5G 1L7; 8 Departments of Biochemistry and Molecular Genetics, University of Toronto, Toronto, Ontario, Canada M5S 1A8; 9 Department of Biology, University of Pennsylvania, Philadelphia, Pennsylvania 19104, USA; 10 Department of Pathology, University of Cambridge, Cambridge CB2 1QP, UK; 11 Biological Resource Center for Toxoplasma, INSERM, University Limoges, CHU Limoges, UMR_S 1094, Tropical Neuroepidemiology, Institute of Neuroepidemiology and Tropical Neurology, Limoges 87025, France; 12 Department of Microbiology and Immunology, Stanford School of Medicine, Stanford, California 94305, USA; 13 Department of Biological Sciences, Dietrich School of Arts and Sciences, University of Pittsburgh, Pittsburgh, Pennsylvania 15260, USA; 14 Animal Parasitic Diseases Laboratory, Beltsville Agricultural Research Center, Agricultural Research Service, USDA, Beltsville, Maryland 20705, USA; 15 Department of Veterinary Pathology and Microbiology, Washington State University, College of Veterinary Medicine, Pullman, Washington 99164, USA; 16 Department of Preventive Veterinary Medicine and Animal Health, Faculty of Veterinary Medicine, University of São Paulo, São Paulo, SP CEP 05598-270, Brazil; 17 Departments of Medicine, Pathology, and Microbiology and Immunology, Albert Einstein College of Medicine, Bronx, New York 10461, USA; 18 Department of Pathology, Microbiology & Immunology, University of California, David, California 95616, USA; 19 Department of Microbiology, University of Tennessee, Knoxville, Tennessee 37996, USA; 20 Departments of Molecular Medicine and Global Health, Florida Center for Drug Discovery and Development (CDDI), University of South Florida, 3720 Spectrum Boulevard, Suite 304, Tampa, Florida 33612, USA; 21 State Key Laboratory of Veterinary Etiological Biology, Key Laboratory of Veterinary Parasitology of Gansu Province, Lanzhou Veterinary Research Institute, Chinese Academy of Agricultural Sciences, Lanzhou, Gansu Province 730046, China; 22 Department of Veterinary Science, University of Kentucky, Lexington, Kentucky 40546, USA; 23 Department of Statistics, University of Georgia, Athens Georgia 30602, USA; 24 Institute of Bioinformatics, University of Georgia, Athens, Georgia 30602, USA

## Abstract

*Toxoplasma gondii* is among the most prevalent parasites worldwide, infecting many wild and domestic animals and causing zoonotic infections in humans. *T. gondii* differs substantially in its broad distribution from closely related parasites that typically have narrow, specialized host ranges. To elucidate the genetic basis for these differences, we compared the genomes of 62 globally distributed *T. gondii* isolates to several closely related coccidian parasites. Our findings reveal that tandem amplification and diversification of secretory pathogenesis determinants is the primary feature that distinguishes the closely related genomes of these biologically diverse parasites. We further show that the unusual population structure of *T. gondii* is characterized by clade-specific inheritance of large conserved haploblocks that are significantly enriched in tandemly clustered secretory pathogenesis determinants. The shared inheritance of these conserved haploblocks, which show a different ancestry than the genome as a whole, may thus influence transmission, host range and pathogenicity.

## Abstract (web summary)

*Toxoplasma gondii* is a parasite that causes zoonotic infections in humans. Here, the authors identify tandem amplification and diversification of secretory pathogenesis determinants in the *T. gondii* genome and show that clade-specific inheritance of conserved haploblocks enriched for these determinants shapes population structure.

## Graphical abstract

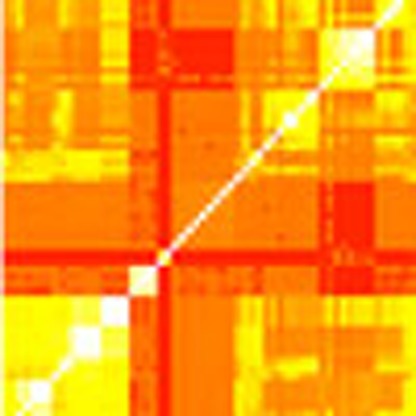

Most of the diversity of eukaryotic life is contained in early branching, unicellular organisms that differ substantially from model organisms such as yeast, flies, worms and mice^1^. This diversity is illustrated by the protozoan phylum Apicomplexa, estimated to contain more than 5,000 species^2^, most being parasitic on insects and mollusks, while a few cause disease in domestic animals and/or humans^3^. Studies of these few disease-causing agents comprise our limited knowledge of this phylum, which demarcate a deep branching phylogeny that has been estimated to span more than ∼400 my of evolution^4^ (Fig. 1a). Over that time frame, it is likely that apicomplexans have adapted to their various vertebrate hosts via multiple independent changes in host range, and yet the molecular mechanisms underlying these adaptations remain largely undefined.

Although most members of this phylum are adapted to a narrow range of hosts, *Toxoplasma gondii* stands out as a generalist. The genus is characterized by a single species that enjoys worldwide prevalence in animals including humans^5^. Infections with *T. gondii* are common^6^, yet they typically only cause disease in immunocompromised hosts, or as a result of transplacental infection^7^. *T. gondii* is equipped with excellent forward and reverse genetic tools, providing a model for many less-tractable apicomplexan parasites^8^. As a highly successful parasite, *T. gondii* is positioned to inform us about genomic features that are important for efficient transmission and expansion of host range. Here, we sought to exploit this potential by analysing the composition and diversity of the *T. gondii* genome in comparison to several closely related apicomplexan parasites.

*T. gondii* belongs to the tissue-cyst forming coccidian parasites, which is distinguished from enteric coccidian parasites by having an alternating two-host (heteroxenous) life cycle (Fig. 1a, Table 1)^5^. Most tissue-cyst forming coccidian parasites have obligatory heteroxenous life cycles (that is, *Sarcocystis* spp. and *Hammondia* spp.), while others share this mode but have evolved additional strategies for transmission (Table 1)^3^. Notably, both *T. gondii* and *Neospora caninum* can cause congenital infection, while only *T. gondii* can be transmitted between intermediate hosts by oral ingestion of infected tissues^9^, thus bypassing the sexual phase of the life cycle. These flexible features in the *T. gondii* life cycle likely aid in transmission through the food chain, thus underlying its broad host range (Table 1). In contrast to our appreciation of differences in life cycle, modes of transmission and host range among these closely related parasites, their molecular bases remain largely unexplored.

In North America and Europe, the population structure of *T. gondii* is dominated by three prevalent clonal lineages^10^, which coexist with much more rare, genetically diverse isolates. A fourth clonal lineage is largely confined to North America, where it is more common in wild animals^11^. In contrast, much greater genetic diversity is seen in South America where the population lacks signs of the recent genetic bottleneck and clonal structure seen in the Northern Hemisphere^10^. *T. gondii* utilizes rodents and birds as natural intermediate hosts, and hence it is particularly well adapted for survival in these niches^3^. Forward genetic mapping studies have identified several families of secretory proteins in *T. gondii* that are important for thwarting innate immunity and hence facilitating infection in the mouse^12^. Related effectors are conserved in *Hammondia hammondi*^13^, hence the basis for the dramatic differences in biology of these two parasites remains unclear. Nonetheless, one hypothesis advanced by the study of select laboratory strains in the mouse model is that pathogenicity, and perhaps host range, may depend on the repertoire of such secretory pathogenicity determinants, although this has not been tested on a wider level.

Here we tested the generality of this hypothesis through genomic analyses of 62 strains of *T. gondii* in comparison to several closely related parasites. Our findings reveal that expansion and diversification of secretory pathogenesis determinants (SPDs), which are often tandemly clustered, is a prominent feature of the genomes of *T. gondii* and related tissue-cyst forming coccidians. Furthermore, patterns of block inheritance, due to recent admixture or selective retention, may underlie specific traits that are shared by related lineages of *T. gondii* containing similar combinations of SPDs. These features define the population structure of *T. gondii* and have implications for the evolution of transmission, host range and pathogenicity.

## Results

### Comparative genomics of tissue-cyst forming coccidians

We undertook a comparative genomics approach to understand the population diversity of *T. gondii* and its relationship to closely related tissue-cyst forming coccidian parasites. First, we generated additional genomic DNA sequence coverage (∼26 × coverage) and RNA-seq data (>1,000 × mean coverage of coding sequence) to improve the assembly and annotation for the reference ME49 strain of *T. gondii* (Table 2). We also generated a whole-genome sequence for *H. hammondi* (∼66 × coverage; Table 2) and compared these two closely related parasites to the recently completed genomes of *Sarcocystis neurona*^14^ and *N. caninum*^15^ (Table 2), which cause economically important diseases in horses and cattle, respectively (Table 1). Finally, to provide insight into genetic variation of *T. gondii* we derived whole-genome sequences for 61 additional isolates that were chosen to span presently known global diversity^16^ (Supplementary Data 1). Among the total of 62 *T. gondii* strains, 16 reference strains representing the major haplogroups were sequenced by both 454 (3 and 8 kb paired-end libraries) and Illumina (300 bp paired-end libraries) technologies and the resulting reads were assembled and annotated separately (∼47 × average sequence coverage) (Supplementary Table 1). The remaining strains were sequenced using Illumina only (∼42 × average sequence coverage) and were aligned to the reference strain ME49 (Supplementary Data 1). Below, we present the comparative analyses of these genomes focusing on three broad themes: (1) comparison of *T. gondii* to the most closely related tissue-cyst forming coccidian parasites, (2) analysis of the core genome of *T. gondii* and how it has diversified and (3) examination of how the global population structure of *T. gondii* has been shaped by local genomic admixture.

**Figure 1:**
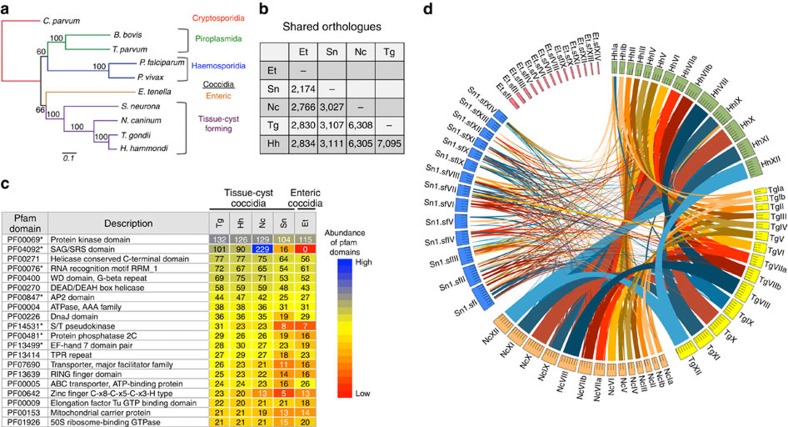
Comparative genomics of tissue-cyst forming coccidian parasites. (**a**) Phylogenetic tree of selected apicomplexans based on a conserved DEAD box helicase protein (TGME49_249810, OrthoMCL OG5126701). Neighbour-joining tree with bootstrap values indicated. Distance equals 0.1 amino acid substitutions/site. Taxa names from http://tolweb.org/Apicomplexa. (**b**) Summary of conserved orthologues based on OrthoMCL analysis. Et, *E. tenella*; Hh, *H. hammondi*; Nc, *N. caninum*; Sn, *S. neurona*; Tg, *T. gondii*. (**c**) Abundance of Pfam domains in coccidian parasites. For each species, the incidence of Pfam domains per protein was determined. The top 20 Pfam domains in *T. gondii ME49* are shown along with the number of proteins containing these domains in each of the species. The cells in the table are colour coded based on the rank of Pfam domains in terms of their abundance. The domains of interest are indicated by an asterisk. Taxa as in **a**. (**d**) Circos plot illustrating levels of synteny among the coccidian parasites. The large outer circle represents the annotated chromosomes or scaffolds of each coccidian species. For *T. gondii*, *H. hammondi* and *N. caninum* all assembled chromosomes (*n*=14) are plotted. For *S. neurona* and *E. tenella*, the largest 14 scaffolds are plotted. Each chromosome/scaffold is labelled with the genus-species abbreviation followed by the chromosome/scaffold number. Tick marks on the chromosome/scaffold represent 1 Mb. The coloured bands and lines linking chromosome/scaffold pairs represent syntenic blocks (minimum of three genes) shared by the chromosomes that are connected. The syntenic links are drawn with *T. gondii, H. hammondi* and *N. caninum* as the reference, in that order. Syntenic blocks were generated using genes present in orthologue clusters, where the cluster contained at least one gene from each species.

We compared the whole-genome sequences from four related tissue-cyst forming coccidian parasites spanning a range of biological hosts and life-cycle strategies (Tables 1 and 2, Fig. 1). Three of the four organisms have a similar total genome size of 62–65 Mb (*N. caninum*, *T. gondii* and *H. hammondi*), while the *S. neurona* genome is somewhat larger due to expanded repeats and much larger introns (Table 2, Supplementary Table 1)^14^. All four genomes have roughly similar GC compositions and are predicted to encode from 7,000 to slightly more than 8,000 genes located on 14 chromosomes, as verified in *T. gondii*^17^ (Table 2). Similar to other genome sequencing projects, 42–56% of the predicted CDSs (coding DNA sequences) encode genes with a putative functional domain annotation, while 44–58% are hypothetical unknowns. To identify conserved features, we compared the four different genomes to the enteric coccidian *Eimeria tenella*^18^ using OrthoMCL to cluster genes into putative orthogroups^19^. Not surprisingly, more closely related taxa showed a higher degree of shared OrthoMCL clusters (Fig. 1b, Supplementary Data 2). Orthogroups classified by Pfam^20^ domains and grouped into the top 20 Gene Ontology (GO) terms (http://geneontology.org/) revealed that all five species share similar orthologous groups for many key biological functions, and that tissue-cyst coccidians are enriched in processes involved in protein modification (Supplementary Fig. 1).

The most abundant protein domains in tissue-cyst forming coccidian parasites include serine/threonine (S/T) kinases, RNA-binding proteins, PP2C-type S/T phosphatases and calcium-binding motifs (EF-hands) (Fig. 1c). There is a precedent for the importance of S/T kinases^21^, such as the expanded polymorphic family of rhoptry (ROP) kinase virulence determinants in *T. gondii*^12^ and of calcium-binding motifs, including within a family of calcium-dependent protein kinases^22^. However, the abundance of RNA-binding proteins (RMR and RMA motifs) was unexpected, as these have been largely unexplored in *T. gondii* and closely related parasites. In addition, plant-like AP2 transcription factors are abundant in tissue-cyst forming coccidian parasites (Fig. 1c), consistent with these being major transcription factors in apicomplexans^23^. Also prevalent in *N. caninum*, *T. gondii* and *H. hammondi* are a family of surface antigens (SAG) called the SRS family (Fig. 1c), which are amplified and highly divergent among tissue-cyst forming coccidian parasites^24^. These appear less abundant in *S. neurona* and absent in *E. tenella* (Fig. 1c), although this result is likely due to their divergence from canonical SRS domains, as similar families of 6-Cys rich proteins occur in other coccidian parasites^18^ and a related family is found in *Plasmodium*^25^. SRS proteins and related 6-Cys proteins share a common extracellular structural domain^26^, are typically GPI-anchored, and are thought to play diverse roles in cell attachment, invasion and development.

From the predicted proteomes, we also reconstructed common metabolic pathways, which were highly conserved across *T. gondii*, *H. hammondi* and *N. caninum*, as noted previously^15^. Expanding this analysis to include the 16 reference strains of *T. gondii* identified paralogues for certain functions, for example, in the pyrimidine and purine metabolic pathways and fatty-acid biosynthesis (Supplementary Fig. 2). Most enzymes involved in energy metabolism were well conserved with few paralogues and/or non-synonymous polymorphisms. Previous studies have established the ability of these pathways to mediate strain-specific growth differences^27^. It is therefore interesting to note that several enzymes with the capacity to modulate flux within these pathways were associated with paralogous expansions and/or significant numbers of non-synonymous polymorphisms (Supplementary Fig. 2, Supplementary Data 3).

Finally, we compared the position of genes across the chromosomes to establish the extent of synteny (Fig. 1d, Supplementary Fig. 3, Supplementary Table 2). There was a high degree of conservation of chromosomal position of orthologous genes between *T. gondii* and *H. hammondi*, and this only slightly decreased when they were compared with *N. caninum*, as previously reported for comparisons of *T. gondii* and *N. caninum*^15^,^28^ (Fig. 1a). Analysis of the more complete *H. hammondi* genome provided here revealed that it shares 29 long syntenic blocks with *T. gondii* harbouring >80% of its genes, with only a few blocks rearranged, most notably a ∼1 Mb reciprocal translocation between chromosomes Ia and IX (Fig. 1d). In contrast, synteny broke down substantially when these three organisms were compared with *S. neurona* and was completely absent when compared with *E. tenella*, as has been described previously for a pairwise comparison of *T. gondii* and *E. tenella*^18^ (Fig. 1d). The loss of synteny since the divergence of enteric from tissue-cyst forming coccidians stands in stark contrast to the conservation of synteny in the kinetoplastidae, fungi and chordates, all groups with greater evolutionary divergence times relative to the coccidians^28^.

### Expanded SPDs in *T. gondii*

**Figure 2:**
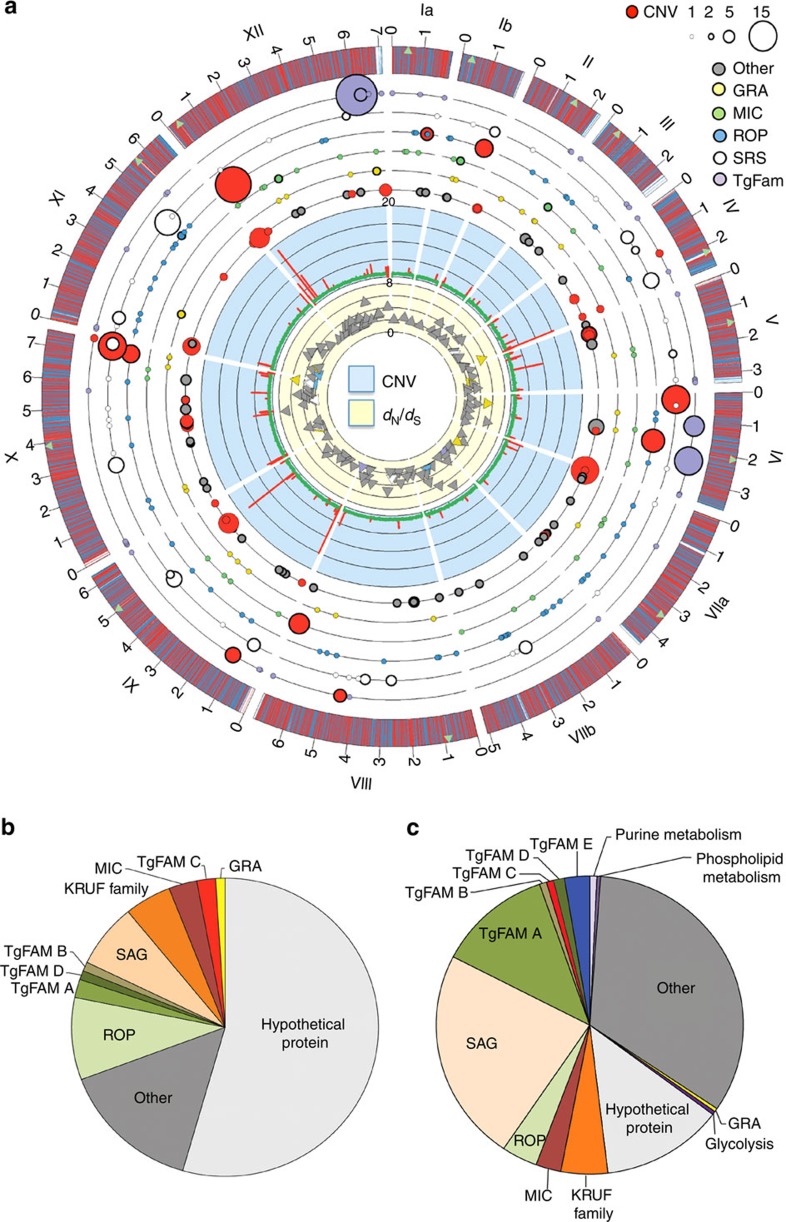
Composition of the *T. gondii* genome. (**a**) Circos representational plot of the *T. gondii* genome based on the reference strain ME49. The outside track plots the position of genes on the 14 chromosomes (numbered outside in Roman numerals with size intervals given in Arabic numerals): top strand gene (blue), bottom strand gene (red), centromere locations (green triangles). The innermost track with yellow background is a scatter plot of genes with *d*_N_/*d*_S_ ratio ≥2 (136 genes): *y* axis 0–8, colours indicate gene family. The second innermost track with blue background is a histogram of regions with copy number variation (CNV) in ME49 using a rolling window of 2,500 bp: *y* axis 0–20, 1X copy level (green), region with CNV (red). The next six circular tracks indicate gene families with tandem duplicates or CNV. From the inside to outside, these six levels represent the following categories: other (grey), *GRA* (yellow), *MIC* (green), *ROP* (blue), *SRS* (white) and T*gFAM* (purple) genes. The size of the circle is relative to the number of genes (range 1–15). Circles with a thick border indicate tandem arrays while red circles indicate CNV. (**b**) Frequency of different gene families among genes with CNV includes SPDs such as *SAG*, *ROP*, *MIC* as well as *TgFAM* genes. (**c**) Frequency of different gene families among genes with tandem duplications in the assembled genomes includes SPDs such as *SAG*, *ROP*, *MIC* and *TgFAM* genes.

To highlight key features of the *T. gondii* genome we depicted the coding capacity of the reference ME49 strain as a Circos plot, where the outermost circle indicates the genes encoded by each of the 14 chromosomes (Fig. 2a, Supplementary Fig. 4). By comparing the average sequence read depth across the genome, we identified chromosomal genes with copy-number variation (CNV) (Fig. 2a, second innermost circle). Expanding this analysis to all 62 strains revealed 14 genes that have evidence of CNV in all strains, and 39 genes with CNV in 90% of the strains (Supplementary Fig. 5, Supplementary Data 4). Examination of patterns of CNV also revealed several examples of large segmental duplications or aneuploidy in specific strains (Supplementary Fig. 6), similar to reports from previous genetic crosses^29^,^30^. These regions were genetically homogeneous suggesting they arose by duplication events and are not hybrids created by unequal crossing over at meiosis. The significance of these diploid regions is uncertain, although recent studies in yeast indicate that aneuploidy can accelerate evolutionary adaptation^31^.

**Figure 3:**
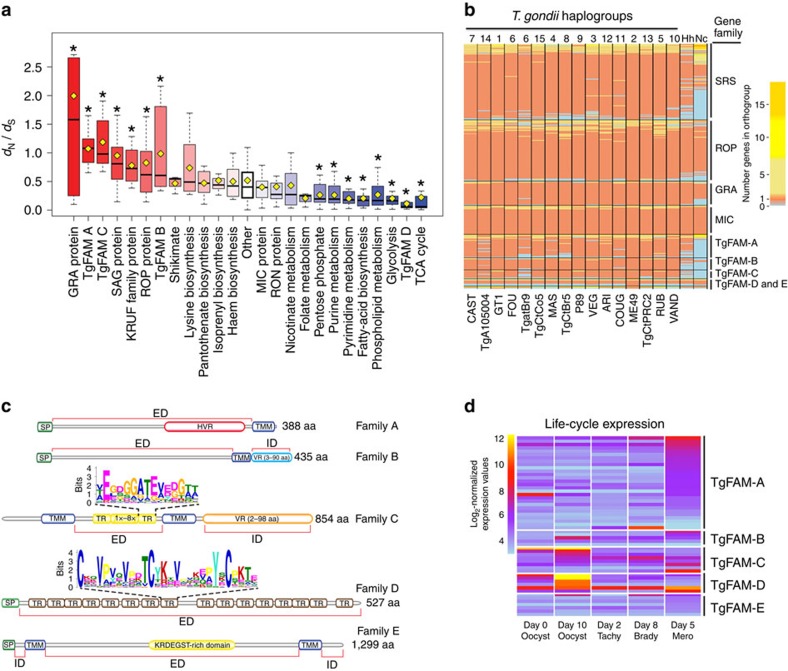
Analysis of expanded or positively selected gene families in *T. gondii*. (**a**) Analysis of positive selective pressure among *T. gondii* gene families based on *d*_N_/*d*_S_. Red values indicate categories with significantly elevated *d*_N_/*d*_S_ ratios, indicating positive selection for diversification, while blue values indicate categories with significantly reduced values, indicative of selection for conservation. Analysis based on 16 reference *T. gondii* genomes, although similar patterns were also seen for all 62 strains. Horizontal black line *d*_N_/*d*_S_ median; yellow square, *d*_N_/*d*_S_ mean; mean values significantly different than the category ‘Other' (*P*≤0.05, Mann–Whitney–Wilcoxon test) are denoted with ‘*'. (**b**) Heatmap of the abundance of SPDs in conserved orthogroups. The number of genes in orthogroups containing SPD genes was determined across the 16 *T. gondii* reference genomes, *H. hammondi* and *N. caninum*, and plotted as a heatmap. Each row is an orthogroup, and the colour of each cell represents the number of genes in that orthogroup, range 0–19. (**c**) Schematic representation of domain structure of *T. gondii* protein families A to E. Family names and average protein lengths per family are indicated on the right. HVR, hypervariable region; KRDEGST-rich domain, protein domain rich in polar amino acids Lysine (K), Arginine (R), Aspartate (D), Glutamate (E), Serine (S), Threonine (T) and Glycine (G); SP, signal peptide; TMM, transmembrane domain; TR, tandem-repeat domain; VR, protein domain of variable length and sequence. Sequence motifs for each tandem-repeat unit are depicted in Logo format. Extra (ED) and intracellular (ID) protein domains, as predicted by Phobius, are indicated below protein schemes. (**d**) Life-cycle expression of *TgFAM* genes. Heatmap of log_2_-normalized microarray expression values for *TgFAM* genes across the *T. gondii* life cycle^36^,^66^,^67^. Background values for the array are near log_2_ of 5. Samples for unsporulated oocysts (Day 0 Oocyst), sporulated oocysts (Day 10 Oocyst), intermediate host tachyzoites (Day 2 Tachy), intermediate host dormant bradyzoite cysts (Day 8 Brady), and enteric stage merozoites (Day 5 Mero) are plotted.

Recent comparisons of the draft genomes of *T. gondii* and *H. hammondi*, and the published genome of *N. caninum*, highlighted the expansion of gene families that differ between these otherwise closely related species^15^,^32^. Using the newly annotated assembly of the ME49 genome obtained here, and data from 61 additional genomes of *T. gondii*, we expanded these analyses to examine the distribution of amplified genes, evident either as CNV or tandem arrays in the assemblies. These amplified genes were plotted as concentric coloured circles corresponding to the protein families they belong to and using symbols proportional to their total copy number (Fig. 2a). Many of these amplified genes encode secretory or surface proteins that have been previously implicated in host pathogenesis, referred to here as SPDs. These SPDs include genes encoding secretory proteins found in micronemes (MICs), dense granules (GRA), ROPs, as well as the SRS super family (Fig. 2a, Supplementary Fig. 4). Members of these protein families are known to mediate host cell attachment (MICs)^33^, modification of host immunity (GRA and ROP proteins)^12^ or adherence and immune evasion (SRS)^24^. Within the ME49 reference genome, we detected a total of 57 gene loci with CNV, which contain 176 gene copies, and 95 loci in tandem arrays, which contain 264 gene copies (Supplementary Data 4). Both CNV and tandemly duplicated genes were enriched in SPDs, in particular in genes encoding SAG/SRS, ROP and MICs (Fig. 2b,c, Supplementary Data 4), a pattern also noted previously^32^. For example, SPDs comprised 52 (35%) of the 152 expanded loci and 196 (45%) of the 440 expanded gene copies, despite making up only 375 (4.5%) of the 8,311 total genes in the ME49 genome. Many of the SPDs also show evidence of positive selection, evident in elevated frequencies of non-synonymous (*d*_N_) versus synonymous (*d*_S_) mutations (Fig. 3a). Among these, the *GRA*, *ROP* and *SAG* genes show some of the highest levels of *d*_N_/*d*_S_, while metabolic enzymes typically show selection for conservation, as seen by low levels of *d*_N_/*d*_S_ (Fig. 3a, Supplementary Data 5).

We expanded the analysis of SPDs to examine their diversity among a set of reference genomes representing the 16 major haplogroups (Fig. 3b). OrthoMCL clustering of the SPD families revealed that while most members were represented in all 16 haplogroups, differences in representation and copy number were most evident in the SRS and ROP families (Fig. 3b). Collectively, these analyses reveal that the major difference between *T. gondii* strains is the diversification of SPD family members.

Comparison of orthologues for *GRA*, *ROP* and *SRS* genes between *T. gondii, H. hammondi* and *N. caninum* revealed substantial differences in clustering by OrthoMCL, suggesting that the divergence among these genes may underlie biological differences between these species (Fig. 3b, Supplementary Fig. 7). In contrast, *MIC* genes were highly conserved, suggesting these organisms use a similar repertoire of host receptors (Fig. 3b). Comparison of OrthoMCL groupings also identified a number of putative species-specific genes unique to *N. caninum, H. hammondi or T. gondii* (Supplementary Fig. 8a, Supplementary Data 6). Further analysis indicated that a subset of the putative species-specific genes represent distant orthologues that are classified as separate groups by OrthoMCL (Supplementary Fig. 8b, Supplementary Data 6). Notably, this distant orthologue category is greater when comparing *N. caninum* to either *T. gondii* or *H. hammondi*, versus the pairwise comparison between the later two species. Among these distantly related orthologues, a number encode *TgFAM* or *SRS* genes, consistent with the idea that they influence important aspects of the biology (Supplementary Data 6). In contrast, a large number of the genes that differ between *T. gondii* and *H. hammondi* show evidence of alternative gene models, including early truncations, premature stop codons and frame shifts (Supplementary Fig. 8b, Supplementary Data 6). In addition, a smaller number of genes were present only in one species and are predicted to be unique, the majority of which were annotated as hypothetical unknowns. Analysis of alternative allele frequencies, RNA-seq data, and sequencing depth coverage failed to find evidence that these predicted differences are due to sequencing or assembly errors and instead suggest that many are genuine (Supplementary Fig. 8 c–f). Consequently, the putative unique gene list provided in Supplementary Data 6 provides a tentative starting point to identify genes that may mediate important biological differences between these closely related species.

In addition to the previously recognized SPDs, we identified families of genes that are uniquely enriched in the *T. gondii* genome, referred to here as *TgFAM* genes (Fig. 3c, Supplementary Data 7), including one previously referred to as Toxoplasma-specific family (*TSF*^18^), which corresponds to *TgFAMC* here. Our analysis of multiple *T. gondii* genomes reveals a much broader set of Toxoplasma-specific families (TgFAMs) (Supplementary Data 7), five of which we have specifically highlighted for their unique domain structures (Fig. 3c). Several TgFAMs are expanded and show evidence of CNV and/or tandem duplication, while others are located at the ends of chromosomes (Fig. 2), as previously noted for the *TSF* family^34^. This pattern of telomeric clustering has also previously been associated with antigenic variant surface adhesins in *Plasmodium*^35^. Although *T. gondii* is not known to undergo antigenic variation, the variable domains of the TgFAMs may represent adaptations to enhance host cell recognition and/or escape immune detection. We have highlighted five of the TgFAMs here based on the fact that they contain conserved signal peptides as well as domain architectures that suggest they may encode surface proteins with extracellular domains that contain conserved protein motifs (Fig. 3c). *TgFAM* genes are expanded in *T. gondii*, although they are less common in *H. hammondi* and *N. caninum* (Fig. 3b, Supplementary Data 7). In particular, *H. hammondi* and *N. caninum* contain far fewer members of *TgFAMA* and *TgFAMB*, and *TgFAMC* appears to be largely absent in *N. caninum* (Fig. 3b, Supplementary Fig. 7). Notably, many of the *TgFAM* genes highlighted here are expressed during sexual development in the cat gut or in oocysts that are shed into the environment following the sexual phase (Fig. 3d)^36^, suggesting they may play roles during transmission. In addition to the *TgFAM* genes highlighted here, there are a number of other gene families containing parasite-specific motifs that are expanded in *T. gondii*, and which may contribute to important biological traits not yet identified (Supplementary Data 7).

### Co-inheritance of haploblocks shape population structure

**Figure 4:**
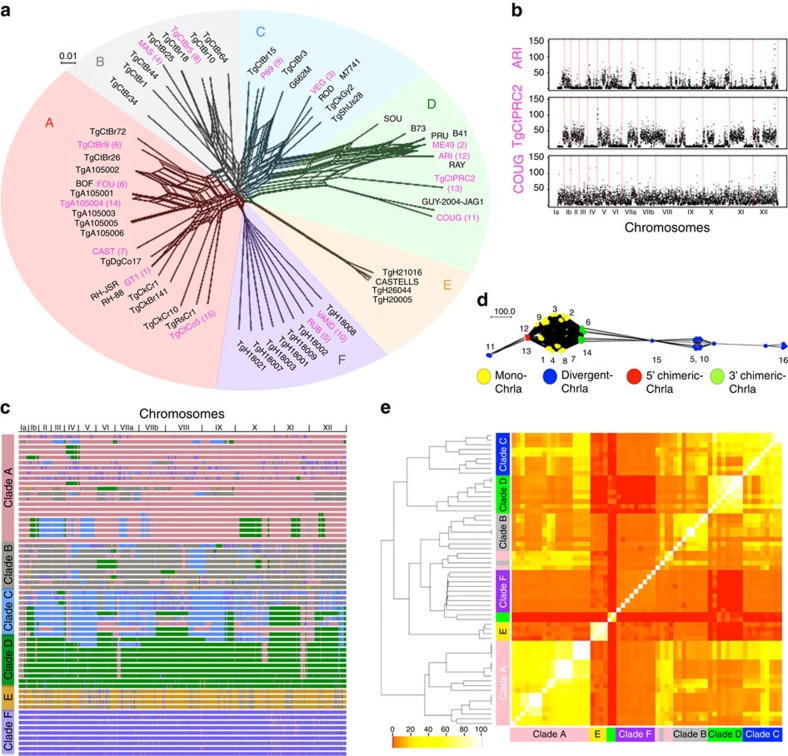
Comparative genomics and population structure of *T. gondii*. (**a**) Population genetic structure of *T. gondii*. Neighbour-net analysis based on genome-wide SNPs (802,764 common data points) from 62 isolates of *T. gondii*. Colour wheel indicates major clades of *T. gondii*. Haplogroup numbers are indicated within parenthesis based on previous designation. Pink names denote the representative strains. Scale bar, number of SNPs per site. (**b**) Pairwise comparison of SNPs between indicated strains to ME49 shown across 14 chromosomes. *y* axis=number of SNPs/10 kb window. (**c**) Chromosome painting of 62 *T. gondii* strains. Local admixture analyses were conducted on SNP blocks of size 1,000 on each of the 14 chromosomes. For each SNP block, local admixture was used to assign strains to a particular ancestral population. The shared inheritance of blocks across members reveals colour patterns that extend vertically in the plot. For example, several pink regions show strong vertical patterns bifurcating across multiple clades, although the dominant colour is not meant to imply origin. (**d**) Network of chromosome Ia (ChrIa) showing high conservation within most haplogroups (number) and clades. Monomorphic forms (Mono, 3′ Chimeric, 5′ Chimeric) are shared by most lineages, while a few strains are highly divergent. Scale bar, total number of changes. (**e**) Heatmap clustering of co-inheritance of shared blocks. The percentage of shared blocks between two strains was determined for all 62 × 62 pairwise strain comparisons (1,953 non-redundant comparisons): Scale bar, % shared blocks. Hierarchical clustering on percent shared blocks independently grouped the strains by clade.

Previous studies have reported the influence of recombination on the global population structure of *T. gondii*, which shows marked geographic segregation of major haplogroups^16^,^37^,^38^, although the factors shaping these patterns remain unresolved. To examine the population structure based on genome-wide polymorphism data, we analysed single nucleotide polymorphisms (SNPs) that were defined by comparison of 61 *T. gondii* strains to the reference ME49 genome and filtered this set to include positions where reliable data were available for all strains (a total of 802,764 positions in each genome (Supplementary Data 8)). Generation of a neighbour network^39^ for these data revealed that the 62 strains group closely with haplogroups and major clades that were previously defined by lower resolution genotyping (Fig. 4a)^16^. Importantly, similar groupings were defined using admixture^40^ (Supplementary Fig. 9) and principal components analysis (Supplementary Fig. 10). Collectively, these findings support a population structure consisting of a small number of clades that show strong geographic segregation, as described previously^16^,^37^.

Although the neighbour network permits visualization of gene flow along several pathways, it does not fully capture the extent or pattern of local genomic admixture among any given pair of strains. To illustrate this more directly, we generated pairwise SNP diversity plots for three of the haplogroups contained in clade D, comparing them to the reference strain ME49 (Fig. 4b). Strains like ARI (haplogroup 12), a sister group of type 2 that is also found in North America, contain large haploblocks that are similar to ME49 (∼60%), interspersed with regions that are divergent (Fig. 4b), consistent with previous findings that these two groups are closely related^11^. In contrast, TgCtPRC2 (haplogroup 13), which is a common clonal genotype in China^41^, shares fewer regions with ME49 (∼40%) and COUG (haplogroup 11), which represents a rare North American lineage found in wild animals, showed almost no conserved regions with ME49 (<1%) (Fig. 4b). Thus although members of a common clade contain distinct genomic patterns that have arisen by different evolutionary paths, it is striking that many share large conserved haploblocks across their genomes.

To better represent the shared ancestry across strains, we analysed local inheritance patterns using chromosome painting to reveal patterns of local admixture. When strains were aligned by clade, the presence of shared haploblocks across members was evident by common colour patterns (Fig. 4c, Supplementary Fig. 11). These shared regions represent chromosomal haploblocks that show a high degree of shared ancestry, in some cases eroding the boundaries of the clade structure. Noteworthy, this analysis also revealed patterns of local admixture that suggest the occurrence of genetic crosses among strains of different clades, likely favoured by their geographic proximity (Fig. 4c, Supplementary Fig. 11). Similar shared chromosomal haploblocks are also seen in average pairwise plots for SNPs among members of individual clades (Supplementary Fig. 12). Among the most strongly conserved haploblocks is chromosome Ia, which is shared across nearly all clades, with the exception of clades E and F (Fig. 4c,d, Supplementary Fig. 13). The basis for the widespread conservation of chromosome 1a (ref. 37) is uncertain, but recent studies suggest that it may be due to the enhanced transmission in domestic cats^42^.

The analyses presented above suggest that common inheritance of large haploblocks is the major factor in determining the phylogenetic grouping of *T. gondii* strains. To test this model, we performed two types of analysis to cluster strains using the SNP data. First, we analysed SNPs using the linkage model of ChromoPainter in FineStructure^43^ to generate a clustering hierarchy. This model, which combines information across linked markers in a co-ancestry matrix, recreated the clades seen in the previous analysis with several minor exceptions (Supplementary Fig. 14). Separately, we analysed the SNPs using a rolling window method to define an overall similarity index based on how many regions were co-inherited between all pairwise comparisons (1,953 unique comparisons), which produced a highly similar clade structure (Fig. 4e, Supplementary Fig. 15). These analyses reveal that the current population structure is defined by recent genomic admixture, where large chromosomal haploblocks have been inherited in common by members of individual clades. Although recent admixture had previously been suggested by analysing individual regions separately^38^, the present genome-wide analysis of SNPs establishes that this pattern is a defining feature of the population structure of *T. gondii*.

**Figure 5:**
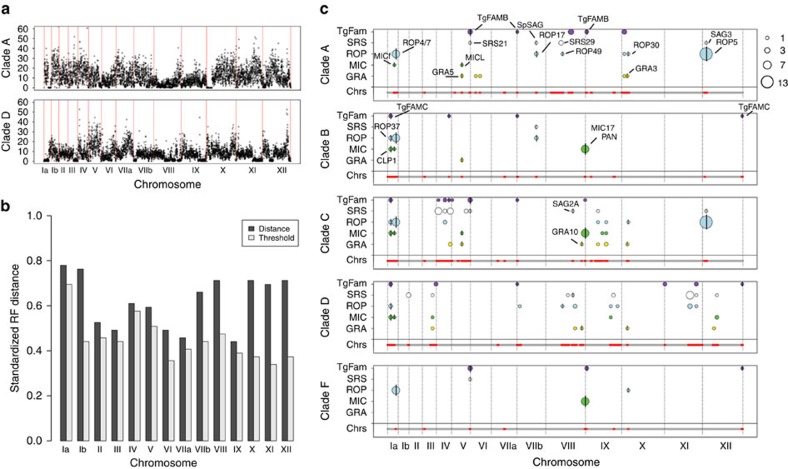
Analysis of conserved regions within and between clades. (**a**) Average number of pairwise SNPs that are shared by members of clade A and clade D, *y* axis 0–60 (outliers >60 not plotted). Each data point represents the average per 10 kb window. Red lines indicate chromosome boundaries. (**b**) Standardized Robinson–Foulds (RF) distances between phylogenetic trees for conserved and non-conserved regions of the genome, based on average pairwise SNP diversity within each clade compared by maximum likelihood. The standardized RF distance equals the proportion of negative branches between the two trees. Threshold distances for statistical significance (*P*≤0.002 per chromosome or ≤0.028 for genome wide) were based on 500 bootstrap trees that were used to establish the variation in tree estimates (the height of the threshold bar represents the 99.998% confidence interval of the tree distance). (**c**) Distribution of conserved regions shared by clades. Horizontal bars below each clade represent the SNP diversity along the combined chromosomes (Chrs). The bar is divided into conserved regions, where the average pairwise SNP diversity is low (red, ≤3 SNPs per 10 kb window), versus non-conserved regions where the SNP diversity is high (grey, >3 SNPs per 10 kb window) based on average pairwise comparisons. Above the bar, the distribution of SPDs within conserved regions for each clade are shown as different colour circles for each gene family: *TgFAM* (purple), *SRS* (white), *ROP* (blue), *MIC* (green) and *GRA* (yellow). Circle sizes are proportional to the number of genes, reflecting tandem clusters or CNV. SPDs that are shared across more than one clade contain a vertical black line and the names are shown in the top-most clade in which they occur. Grey lines indicate chromosome boundaries.

To further examine the pattern of long-haploblock inheritance, we compared the ancestry of regions that were conserved with those that were more variable. When SNP diversity was averaged for members of the same clade, it emerged that discrete regions of the genome show very low SNP diversity, while others are highly variable (Fig. 5a). Regions of low average pairwise SNP diversity were observed in all clades, but differed in their frequency and location (Supplementary Fig. 12). To compare the ancestry of different regions of the genomes, we partitioned the genome into two segments based on regions that exhibited low SNP diversity in at least one clade (defined as the union of all regions that were ‘conserved' in at least one clade) versus regions that showed high SNP diversity in all clades (defined as ‘non-conserved') (Supplementary Data 9 and 10). We then compared unrooted phylogenetic trees for the conserved versus non-conserved regions using a Robinson–Foulds distance metric, which measures the degree of difference, or distance, between the two sets of trees. This analysis revealed that the ancestry of the conserved haploblocks was significantly different than that of the non-conserved regions for all 14 chromosomes (Fig. 5b). In addition, we generated neighbour networks based on the conserved versus non-conserved regions (Supplementary Fig. 16). The conserved region network most closely resembled the total SNP network (Fig. 4a) and the network based on the non-conserved regions grouped most strains in similar clades, with several notable exceptions (Supplementary Fig. 16). These findings illustrate the importance of the conserved blocks in influencing the grouping of genotypes into clades.

To determine the influence of these shared haploblocks on the content of genes found within specific clades, we plotted the distribution of SPDs found within conserved regions shared by members of specific clades (Fig. 5c, Supplementary Data 11). The distribution of SPDs revealed that a number of known pathogenicity determinants were common to conserved regions in specific clades (Fig. 5c). We tested whether these patterns were random, or if they showed specific enrichment of SPDs within conserved regions. When clades A, B, C, D and F were analysed together, the pattern of clustering of SPDs was highly significant (*P*≤0.005) allowing us to reject the null hypothesis that they were randomly distributed across the genome. SPDs were also significantly clustered in conserved regions when separately analysing clade C (*P*≤0.005), clade D (*P*≤0.05), and clade F (*P*≤0.000001), while clade B was suggestive (*P*≤0.08) and clade A was not significant (*P*≤0.2). The failure to observe a significant clustering of SPDs in clade A may be due to its considerable substructure that suggests it may actually be comprised of two or more distinct groups. Nonetheless, it is clear that SPDs are often clustered and are found with increased frequency in conserved, shared regions of the genome. SPDs within these regions also share the recent ancestry of the surrounding conserved regions when analysed using phylogenetic trees (Supplementary Figure 17). These findings are consistent with the hypothesis that recent inheritance of conserved blocks containing specific SPDs is associated with successful expansion of specific lineages and suggest that SPDs impart a selective advantage to members of specific clades.

The co-inheritance of SPDs within conserved regions provides a tentative list of candidates for further study of genes that may underlie important biological traits shared by specific clades. Among these are a number of SPDs previously implicated in acute virulence in the mouse: for example *ROP17* (ref. 44) found in conserved regions in clades A and B, *ROP5* (ref. 29) found in conserved regions in clades A and C, and *GRA3* (ref. 45) found in conserved regions in A, C, D, and F (Fig. 5c). Low diversity regions also contain a number of *SRS* genes, encoding immunologically dominant surface proteins, which have previously been implicated in host cell invasion including sporozoite SAG (Sp-SAG also known as SRS28)^46^ found in conserved regions in clades A and B, SAG3 (SRS57)^47^ found in conserved regions in clades A and C, and SAG2A (SRS34A) (M.E.G., unpublished) found in conserved regions in clades C and D (Fig. 5c, Supplementary Data 11). A second pattern that emerges from this analysis is the presence of clusters of SPDs that are clade-specific, for example clusters of *SRS* and *TgFAM* genes on chromosomes IV, V and IX found in clade C, and clusters of various SPDs on chromosomes II, IX, XI and XII in clade D (Fig. 5c, Supplementary Data 11). Although the specific roles of these genes are unknown, they may underlie common traits that distinguish phenotypes characteristic of specific clades.

## Discussion

*Toxoplasma gondii* belongs to a diverse and ancient phylum of parasites that antedates the wide range of vertebrate hosts that they currently inhabit. It shares a core set of genes and metabolic processes with closely related tissue-cyst forming coccidian parasites. Despite having similar genomic content, these organisms differ dramatically in their host range, pathogenicity and modes of transmission. We demonstrate here that *T. gondii* is demarcated from its closest relatives by the expansion of parasite-specific SPDs that are involved in host–pathogen interactions. Diversification of SPDs also highlights key differences among major clades of *T. gondii*, which are distinguished by common inheritance of large haploblocks in their genomes. Shared inheritance of large haploblocks among related strains reinforces the hypothesis that recombination in the wild, while infrequent, drives important biological adaptations^48^,^49^. The distribution of clustered SPDs within conserved regions that show common ancestry identifies a number of candidate genes that may influence both clade specific and more broadly shared traits. Overall, the phenotypic traits of individual strains are likely determined by both their core ancestral genomes, and inheritance of conserved haploblocks, which together comprise their mosaic genomes.

The mosaic genomic patterns seen in specific clades may underlie differences in population structure that exist in different *T. gondii* populations between North and South America^10^. Although the common ancestry of conserved blocks among otherwise different genotypes is consistent with recent introgression, the conservation of these regions among members of a given clade may reflect several different mechanisms. Such shared haploblocks may be retained in the face of ongoing recombination in outbreeding populations, suggesting they impart a selective advantage. Alternatively, they may simply reflect recent admixture that has not been eroded due to infrequent recombination, such as in clonal populations. Regardless of their exact histories, strains that inherit conserved haplotype blocks in common will also share clusters of highly related genes, including SPDs that may influence traits such as transmission, host range and pathogenesis.

Expansion of polymorphic genes that are important in pathogenicity is also a key feature of other pathogen genomes. One feature they share in common is that the amplified genes typically encode surface or secretory proteins that interact directly with the host, either to mediate attachment or immune evasion. Examples include: the expansion of surface antigen variants encoded by *VAR* genes in the *Plasmodium falciparum* genome^50^, and the unrelated yet expanded *VIR* genes in *Plasmodium vivax*^51^, variant surface glycoprotein encoding genes (*VSG*) in *Trypanosoma brucei*^52^, and the expansion of RXLR effectors in oomycetes^53^. It is noteworthy that while gene expansion and diversification are common to each of these examples, the protein families involved are largely distinct and reflect the specialized biology of these diverse pathogens. This pattern suggests that expansion of polymorphic gene families is a common theme that underlies important changes in host range and transmission that characterize the evolution of pathogens in their diverse hosts.

## Methods

### Propagation of strains and isolation of gDNA

Sixty-two representative strains of *T. gondii* were selected from different haplogroups from around the world (Supplementary Data 1)^16^. Strains were cultured in human foreskin fibroblast cells, as described previously^16^.

### Genome sequencing of *T. gondii* reference strains

Sequencing of 16 reference strains of *T. gondiii*, and of one isolate of *H. hammondi*, was conducted using a combination of 454, and Illumina PE sequencing technologies (Supplementary Table 1). Sequence reads were screened for contamination and reassembled using Celera Assembler software^54^ or Newbler v2.6 (ref. 54). Scaffolds were then aligned with MUMmer^55^ to *T. gondii* ME49 chromosome sequences from ToxoDB v8.0 (http://ToxoDB.org) to generate super-scaffolds spanning entire chromosomes. Annotated genomes were deposited into National Center for Biotechnology (NCBI).

### Genome sequencing for SNP discovery

For each of the remaining 46 non-reference strain (Supplementary Data 1), a single Illumina PE barcoded library was prepared from tachyzoite gDNA. Libraries were then pooled into groups of nine samples and sequenced multiplexed in a single lane of an Illumina HiSeq 2000 machine. Sequencing reads were deposited in the Sequence Read Archive repository at NCBI.

### Sequencing of tachyzoite messenger RNA samples

To aid in the curation of ME49 gene models, two tachyzoite-specific Illumina complementary DNA libraries were constructed from mRNA isolated from tachyzoite cultures from ME49 and GT1 strains. Each library was then sequenced in a single lane of an Illumina Genome Analyzer II machine.

### Structural and functional annotation of the ME49 genome

Gene annotations were derived by comparison of the existing ME49 reference genome (http://ToxoDB.org) using a combination of evidence from RNASeq data, cDNA/EST sequences and a variety of software tools to predict potential protein-coding genes using an in-house pipeline at J. Craig Venter Institute (JCVI) (Supplementary Methods). Predicted proteins were run through JCVI's autonaming pipeline, that assign product names based on a number of sequence similarity searches including blastp searches against the previous *T. gondii* ME49 proteome (ToxoDB v8.0; http://ToxoDB.org) and the GenBank non-redundant protein database, HMM searches against Pfam and TIGRfam^56^ databases, and RPS-Blast searches against the NCBI-CDD database^56^. Proteins without any significant hit to other proteins or protein domains were flagged as ‘hypothetical protein'. The final list of product names was then curated by researchers from the *Toxoplasma* research community before being assigned to working models. ME49 protein-coding genes were assigned similar pub_locus identifiers to the previous genome assembly while newly predicted protein-coding genes were assigned completely new pub_locus identifiers (Supplementary Methods).

### Annotation of *T. gondii* reference strains and *H. hammondi*

Functional annotation of protein-coding genes in other *T. gondii* reference strains was performed as above. Genes syntenic to ME49 inherited their product names, GO terms, and Enzyme Commission numbers, while non-syntenic genes acquired their names and other functional annotations from the output of JCVI's autonaming pipeline. Structural and functional annotations of *H. hammondi* were carried out following a similar approach with slight modifications (Supplementary Methods).

### Domain Identification of *T. gondii* novel gene families

To identify known protein domains the *T. gondii* ME49 proteome was searched against Pfam and TIGRfam HMM profiles using HMMER3 ref. 57). Proteins matching a particular HMM profile were assigned to that domain and remaining peptides searched against each other using blastp to identify potential novel domains. The top five protein families containing novel para domains (TgFAMs A to E) were analysed using Phobius^58^ to identify signal peptides and transmembrane domains. *De novo* identification of conserved protein domains across members of the same gene family was carried out with MEME^59^. Expression levels for the TgFAMs were obtained from *T. gondii* Affymetrix Array data available from NCBI GEO records GSE32427 and GSE51780.

### Estimation of *d*_N_/*d*_S_ ratios

Coding sequences from each cluster of orthologous genes from the 16 *T. gondii* reference strains were used to estimate *d*_N_/*d*_S_ ratios using a modified version of the Bioperl script *bp_pairwise_kaks.pl* (http://search.cpan.org/dist/BioPerl/scripts/utilities/bp_pairwise_kaks.pl).

### SNP identification

Illumina reads for each of the 61 other genomes were aligned using Bowtie2—end-to-end^60^ against the ME49 reference genome assembly (release date 23 April 2013), identifying a total of 2,342,433 SNPs across all strains. Positions with informative base calls for all 62 strains were identified, generating a final list of 802,764 SNPs that were used for analysis.

### Analysis of orthologous genes

Annotated proteomes were analysed using OrthoMCL v2.0 (ref. 61) to define orthologous groups. Clusters of orthologous groups were functionally annotated using GO Slim terms, which are designed to group the many different GO terms into smaller groups of related processes (http://geneontology.org/page/go-slim-and-subset-guide). The proteomes were queried against the Pfam HMM database using HMMER3 to estimate the abundance of Pfam domains.

### Mapping metabolic differences

SNPs from the 16 *T*. *gondii* reference strains that correspond to the 382 proteins in the iCS382 metabolic pathway reconstruction of *T*. *gondii* ME49 (ref. 27) were downloaded from ToxoDB (http://www.toxodb.org/toxo-release4-0/home.jsp).

### Network and principal components analyses

Genome-wide SNPs were saved as FASTA files and directly incorporated into SplitsTree v4.4 (ref. 39) to generate unrooted phylogenetic networks using a neighbour-net method and 1,000 bootstrap replicates. Principal components analysis was performed by eigenanalysis of a co-ancestry matrix implemented in fineSTRUCTURE, as described in ref. 43.

### Chromosome Ia analysis

SNP data for ChrIa were plotted as a minimum spanning tree using SplitsTree v4.4 (ref. 39) with 2,000 spring-embedded iterations. The 62 strains were clustered into four major groups denoted as monomorphic, divergent, 5′-chimeric and 3′-chimeric chromosome Ia. SNPs present in each cluster were calculated using a custom script over a 10-kb moving window and plotted using Excel.

### Admixture analysis

The population genetic structure of *T. gondii* was determined by an unsupervised clustering algorithm, ADMIXTURE^40^ with ancestral clusters set from *k*=1 through 10. The number of ancestral clusters *k* was determined by estimating the low cross-validation error (CV error) for different *k* values using five-fold CV.

### Co-ancestry heatmap

We developed a co-ancestry heatmap by using the linkage model of ChromoPainter (http://www.paintmychromosomes.com) and fineSTRUCTURE^43^ based on the genome-wide SNP data. The burn-in and Markov Chain Monte Carlo (MCMC) after the burn-in were run for 10,000 iterations with default settings.

### Estimating CNV

For each strain of *T. gondii*, the respective.sra files were used to align reads to the 14 ME49 reference chromosomes using Bowtie2 with the end-to-end option. The read depth per base pair, or read bases (RB), across 8,320 chromosomal-mapped genes was determined using samtools mpileup^62^. Plots were generated in R (http://www.r-project.org/). *T. gondii* gene families organized in tandem arrays were identified with an in-house *perl* script.

### Analysis of OrthoMCL species-specific genes

Genes found to be specific to *T. gondii, H. hammondi or N. caninum* based on OrthoMCL clustering were further analysed using a combination of sequence alignment tools (Supplementary Methods). Genes were classified based on whether they showed a significant ‘Blastp hit', showed blastn similarity that was either ‘full length' or constituted an ‘alternative gene model', or showed no similarity and were ‘unique'. Further analysis was done to investigate these differences by RNA-Seq, analysis of minimum alternative allele frequency and minimum read depth (Supplementary Methods).

### Regions of co-inheritance

To determine the extent of recombination and co-inheritance of blocks between strains, low SNP regions (regions of recent co-inheritance or shared blocks) were identified for 10 kb windows for pairwise strain comparisons. A heatmap was generated using the R function heatmap.2 (gplots library (http://www.r-project.org/)) with hierarchical clustering on the % shared blocks value. The number of SNPs per 10 kb window were averaged for all strains within a Clade, and chromosomal regions with low SNP density were identified as above using 10 kb windows that had three or fewer SNPs across a continuous stretch of 10 windows (100 kb), allowing for intermittent outliers.

### Identification of SPD genes and clustering within the genome

We identified genes that belong to the SPD families (that is, *MIC*, *GRA*, *ROP*, *SRS* and *TgFAM*) based on the annotation of ME49 accounting for CNV in determining the gene number. We then mapped the position of the SPDs onto the assembled ME49 genome and defined those that fell into conserved or non-conserved regions. To determine if gene type was independent of region type we compared the observed frequency of SPDs and non-SPD genes in conserved versus non-conserved regions of the genome using a χ^2^-squared analysis. The null hypothesis was that the distribution would be random, and there would be no difference between observed and expected. A *P* value of ≤0.05 was considered significant cause for rejection of the null hypothesis.

### Ancestry of conserved and non-conserved regions

Phylogenetic trees for the conserved and non-conserved regions were constructed using maximum likelihood as implemented in RAxML version 7.3.0 with the GTR+GAMMA model^63^. Standardized Robinson–Foulds distances^64^ were calculated between the conserved and non-conserved trees based on 500 bootstrap replicates. Trees were considered congruent if they had no conflicting branches with bootstrap support of >95%.

### Phylogeny

Phylogenetic trees were constructed for the conserved OrthoMCL OG5_0126701 using the Neighbour-Joining algorithm with 1,000 bootstrap replicates as implemented in Geneious ver. 7.1.5 (http://www.geneious.com (ref. 65)) and visualized with FigTree ver. 1.4.0 (http://tree.bio.ed.ac.uk/software/figtree/).

### Synteny

The OrthoMCL ortholog clusters (see above) were reformatted to represent each pair found in the cluster outside of self-matches and syntenic blocks were generated between all combinations of genomes as described in ref. 28.

### Chromosome painting

Local admixture analyses using an enhanced ADMIXTURE algorithm^40^ was used to assign each of the 62 strains to clusters representing these ancestral states.

### Additional methodology

Detailed methods for the above sections can be found in the supplementary methods.

## Additional information

**Accession codes**: Assembled and annotated sequences for the genomes of the *T. gondii* reference strains have been deposited in GenBank at NCBI under accession codes GT1 (GCA_000149715.2), VEG (GCA_000150015.2), ME49 (GCA_000006565.2), MAS (GCA_000224865.2), RUB (GCA_000224805.2), CAST (GCA_000256705.1), TgCtBr5 (GCA_000259835.1), P89 (GCA_000224885.2), VAND (GCA_000224845.2), COUG (GCA_000338675.1), ARI (GCA_000250965.1), TgCtPRC2 (GCA_000256725.1), GAB2-2007-GAL-DOM2 (GCA_000325525.2), TgCtCo5 (GCA_000278365.1), FOU (GCA_000224905.2) and TgCtBr9 (GCA_000224825.1). Assembled and annotated sequences for the genomes of the *H. hammondi* reference strains have been deposited in GenBank at NCBI under accession code GCA_000258005.2. The annotation of reference genomes are also available at ToxoDB (http://toxodb.org/common/downloads/WhitePaperProvisionalGenomes/): RNA-Seq reads for *T. gondii* strains have been deposited in the Sequence Read Archive at NCBI under accession codes GT1 (SRX099798) and ME49 (SRX099799). Sequence reads from the remaining *T. gondii* strains have been deposited in the Sequence Read Archive at NCBI under the following accession codes RH-88 (SRX160127, SRX160126), RH-JSR (SRX159850, SRX159851), TgCkCr1 (SRX160807), SOU (SRX160119), TgCkGy2 (SRX099785), CASTELLS (SRX099789), TgCatBr1 (SRX099790), TgCatBr18 (SRX099794), TgCatBr34 (SRX160051), BRC TgH 18002 GUY-KOE (SRX099796), BRC TgH 18003 GUY-MAT (SRX099783), GUY-2003-MEL (SRX160131), GUY-2004-ABE (SRX160132), TgRsCr1 (SRX160143), TgCkBr141 (SRX160124), TgCatBr25 (SRX160134), TgCatBr10 (SRX099791), TgCatBr64 (SRX160743), BRC TgH 18001 GUY-DOS (SRX099782), TgCkCr10 (SRX099784), B41 (SRX099774), RAY (SRX099793), TgCatBr44 (SRX160141), GAB1-2007-GAL-DOM10 (SRX159841, SRX159839), GAB3-2007-GAL-DOM9 (SRX160125), GAB5-2007-GAL-DOM6 (SRX159842, SRX159840), TgCatPRC3 (SRX09979), BRC TgH 18009 (SRX171132), BRC TgH 18021 (SRX160041), BRC TgH 20005 (SRX157499, SRX157465), BRC TgH 21016 (SRX099775), GUY-2004-JAG1 (SRX099776), B73 (SRX159844, SRX159843), PRU (SRX099792), M7741 (SRX159890, SRX159849), ROD (SRX160129, SRX160128), TgShUS28 (SRX160130), BOF (SRX099774), GAB5-2007-GAL-DOM1 (SRX160069), TgCatBr26 (SRX099780), TgCatBr72 (SRX160049), TgDogCo17 (SRX099787), TgCatBr15 (SRX099779), G662M (SRX160052), GAB3-2007-GAL-DOM2 (SRX160123), TgCatBr3 (SRX160142) and TgH26044 (SRX160050).

**How to cite this article:** Lorenzi, H. *et al.* Local admixture of amplified and diversified secreted pathogenesis determinants shapes mosaic *Toxoplasma gondii* genomes. *Nat. Commun.* 7:10147 doi: 10.1038/ncomms10147 (2016).

## Supplementary Material

Supplementary Figures, Supplementary Tables, Supplementary Methods and Supplementary References Supplementary Figures 1-17, Supplementary Tables 1-2, Supplementary Methods and Supplementary References

Supplementary Data 1 List of *T. gondii* strains used.

Supplementary Data 2 OrthoMCL clusters for *E. tennella, S. neurona, N. caninum, T. gondii* and *H. hammondi*.

Supplementary Data 3 OrthoMCL clusters for *N. caninum, H. hammondi* and 16 reference genomes of *T. gondii*.

Supplementary Data 4 List of CNV and tandem arrays for 16 reference *T. gondii* genomes.

Supplementary Data 5 List of *T. gondii* genes with top dN/dS ratios.

Supplementary Data 6 Analysis of shared and specific genes for *N. caninum, T. gondii* and *H. hammondi*.

Supplementary Data 7 List of parasite specific gene families

Supplementary Data 8 List of SNPs found in all 62 strains

Supplementary Data 9 Conserved region SNPs

Supplementary Data 10 Non-conserved region SNPs

Supplementary Data 11 Summary of SPDs in conserved regions

## Figures and Tables

**Table 1:** Summary of the life cycle, host range and pathogenicity of tissue-cyst forming coccidian parasites.

**Organism**	**Definitive host**	**Alternative host(s)**	**Transmission**	**Animal disease**	**Human disease**
*Sarcocystis neurona*	Opossum	Skunk, raccoon and other small mammals	Obligatory sexual–asexual cycle	Myeloencephalitis in horses and marine mammals	None
					
*Neospora caninum*	Canine	Bovine	Vertical asexual or sexual–asexual cycle	Congenital, abortion in cattle neurological, paralysis in dogs	None
					
*Hammondia hammondi*	Feline	Rodents	Obligatory sexual–asexual cycle	None	None
					
*Toxoplasma gondii*	Feline	Warm-blooded vertebrates	Vertical asexual, sexual–asexual cycle or direct asexual transmission	Congenital, abortion in sheep	Opportunistic pathogen in humans; CNS, ocular, congenital

**Table 2:** Summary of genome features for *T. gondii* and representative apicomplexans.

**Feature**	S. neurona*	***N. caninum***†	***H. hammondi***‡	***T. gondii*** **ME49**§
Estimated size	∼127 Mb*	∼62 Mb†	∼65 Mb	∼65 Mb
Assembly length without sequencing gaps (bp)	117,871,271	57,524,119	67,460,985	65,464,221
Number of scaffolds||	116	NA¶	99	47
Scaffolds N50 (bp)	2,890,735	NA	1,494,935	6,301,488
Number of contigs#	8,903	241	1,337	410
Contigs N50 (bp)	20,915	405,161	84,429	1,219,553
Sequencing depth	375 ×	8 × †	66 ×	26.5 ×
# Chromosomes	NA	14†	14	14
# Protein-coding genes	7,093	6,936**	8,004‡	8,322§
GC content	51.5%	54.8%	52.5%	52.2%
% Protein-coding sequence††	50.9%	59%	57.3%	60.5%
Average length of protein-coding genes‡‡(bp)	9,121	4,892	4,868	4,778
Average number of exons per protein-coding gene	5.5	12	11.7	11.5

NA, not available.

^*^All sequence reads were deposited in the National Center for Biotechnology (NCBI) sequence read archive under accession SUB554996 ref. 14.

^†^Reid *et al.*^15^.

^‡^GenBank Assembly ID GCA_000258005.2.

^§^GenBank Assembly ID GCA_000006565.2.

^||^Scaffolds >10,000 bp.

^¶^960 Scaffolds, any size.

^#^Contigs >2,000 bp.

^**^GenBank Assembly ID GCA_000208865.2.

^††^Exons and introns, without UTRs.

^‡‡^Without UTRs.
