# Supplementary material for: Local admixture of amplified and diversified secreted pathogenesis determinants shapes mosaic Toxoplasma gondii genomes
Source: Nat Commun. 2016 Jan 7;7:10147. doi: 10.1038/ncomms10147 (PMC4729833; doi:10.1038/ncomms10147)
Supplement: Supplementary Figures, Supplementary Tables, Supplementary Methods and Supplementary References — Supplementary Figures 1-17, Supplementary Tables 1-2, Supplementary Methods and Supplementary References [file ncomms10147-s1.pdf]

Supplementary Figures, Tables, Methods, and References

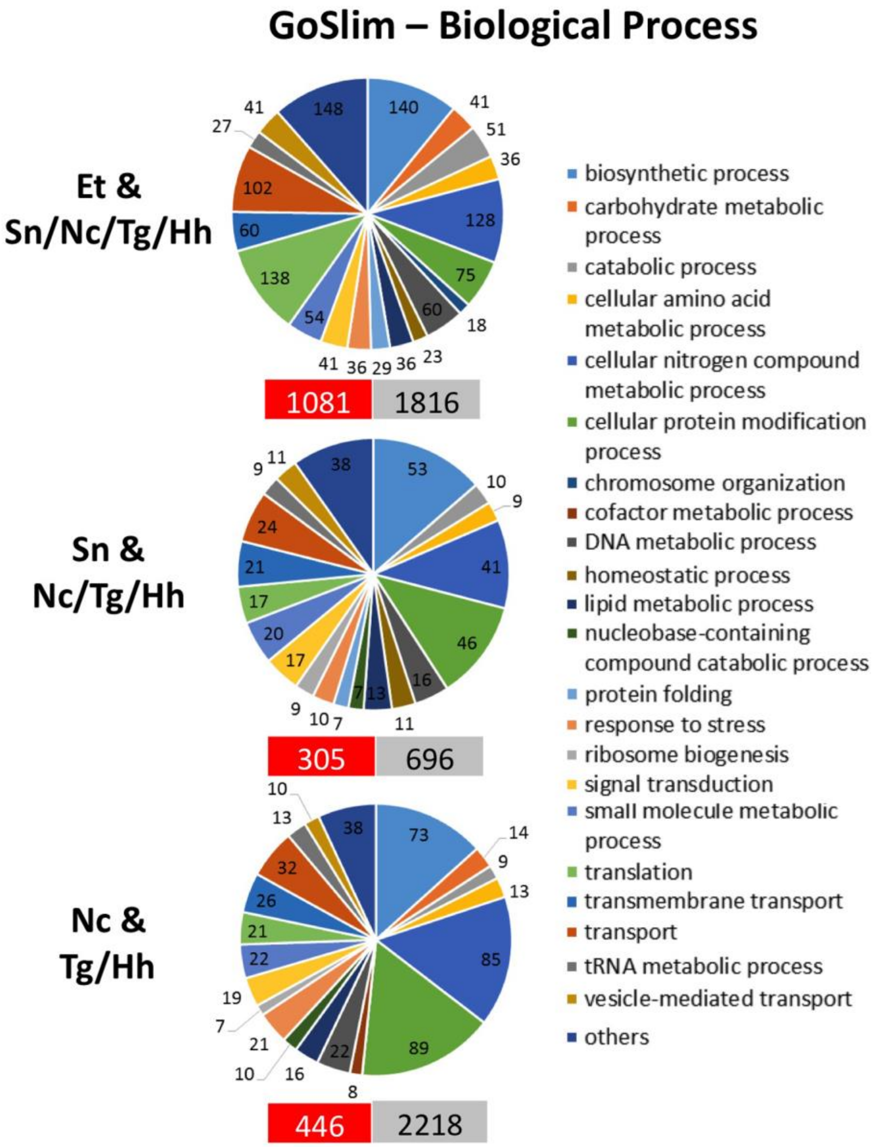

Supplemental Figure 1 Pie-charts representing the top 20 GO Slim annotations (Biological Process) of clusters of orthologous groups. At each branch, OrthoMCL groups present in the outer species and at least one of the internal taxa are included. Numbers in grey indicate orthogroups without pfam domains, numbers in red indicate those with pfam annotations. Et – *E. tenella*, Sn - *S. neurona*, Nc - *N. caninum*, Tg - *T. gondii*, Hh - *H. hammondi*. As expected, all 5 coccidians share orthologous groups corresponding to many key biological processes. However, it is noteworthy to see that the group of tissue-cyst coccidians (Sn ,Nc, Tg, Hh) also share 1,001 orthogroups not present in Et. In particular, they are enriched for processes involved in protein modification. This trend is also observed in the orthogroups shared by the closely related set of Nc, Tg, and Hh (2,664 orthogroups in common), suggesting a key role for processes involving protein modification during their evolution.

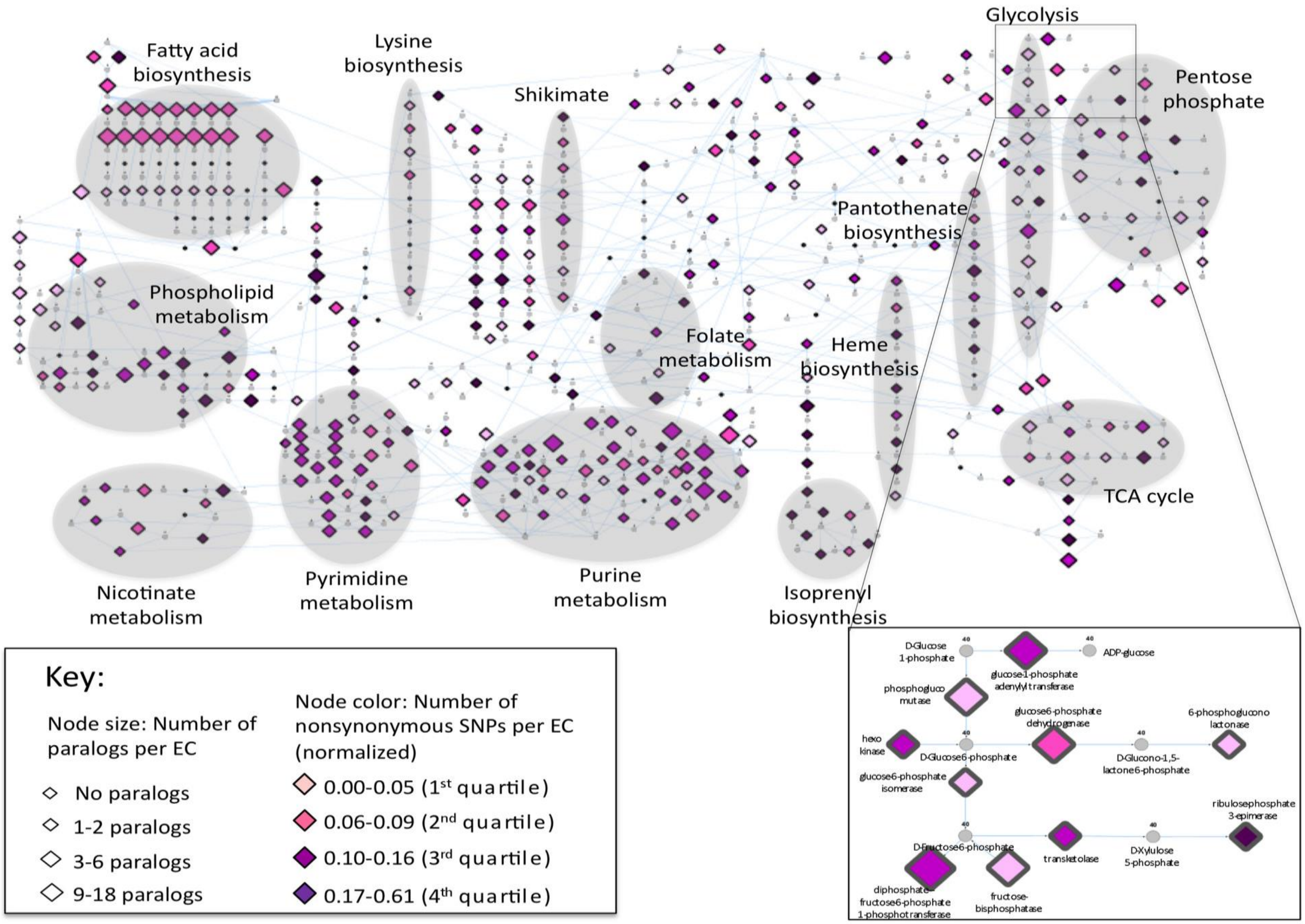

Supplemental Figure 2 Metabolic network of *T. gondii* (iCS382) annotated based on non-synonymous SNPs and number of paralogs. The metabolic network reconstruction of *T. gondii* (iCS382) is shown with nodes representing enzymes (diamonds) and metabolites (circles), connected by edges. Groups of enzymes constituting individual pathways (according to KEGG) are enclosed in grey circles. The number of paralogs for each EC number is represented by node size. The frequency of non-synonymous SNPs (including nonsense SNPs) for each EC number is represented by node color. These values are normalized for the length of the protein and number of paralogs. The region corresponding to the beginning of the energy metabolism pathways glycolysis and pentose phosphate pathway is enlarged and shown as an inset.

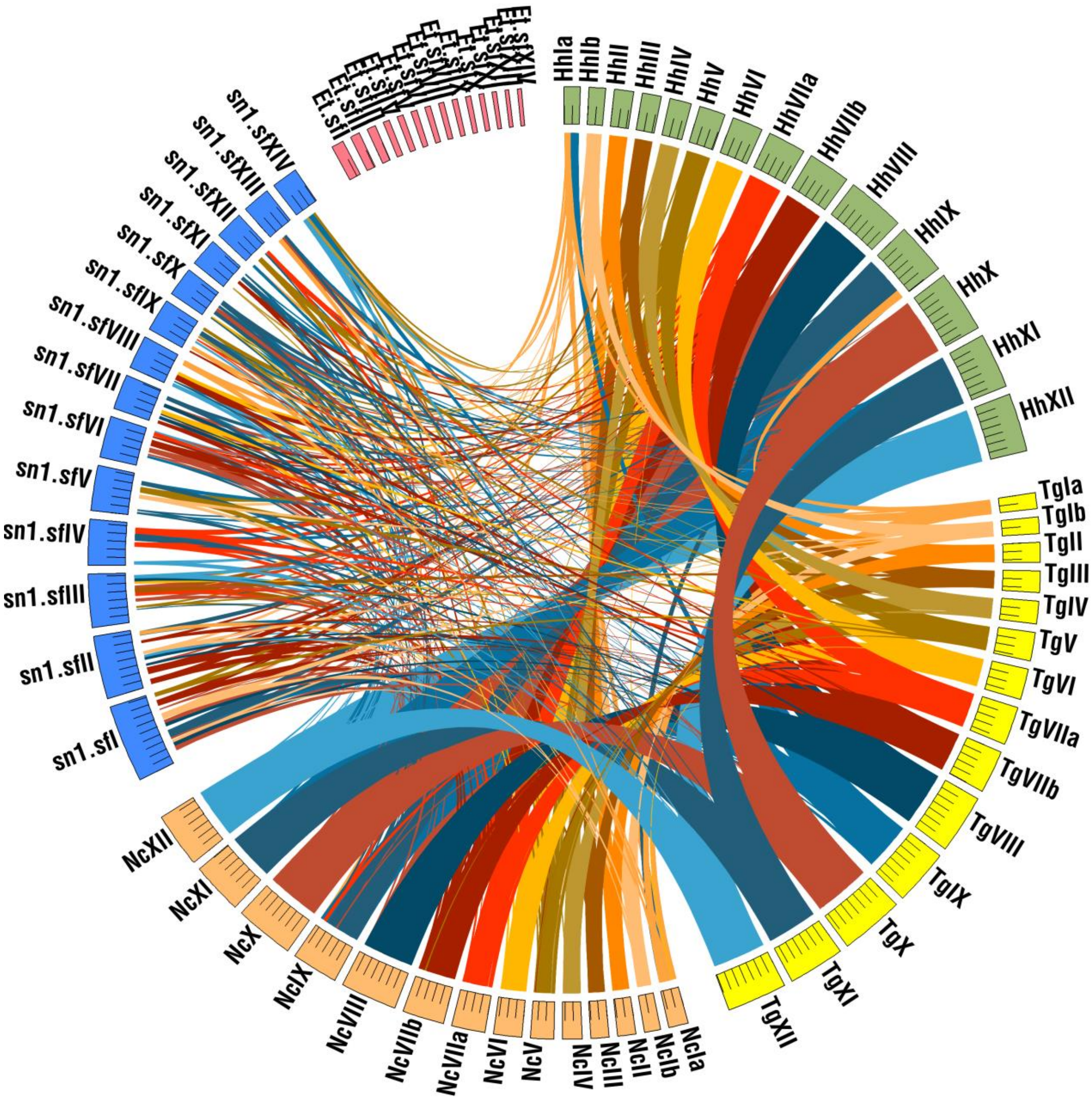

Supplemental Figure 3 Levels of synteny (relaxed criteria) among the coccidia. The large outer circle represents the annotated chromosomes or scaffolds of each coccidian species. For *T. gondii, H. hammondi* and *N. caninum* all assembled chromosomes (n=14) are plotted. For *S. neurona* and *E. tenella* the largest 14 scaffolds are plotted. Each chromosome/scaffold is labeled with the genus-species abbreviation followed by the chromosome/scaffold number. Tick marks on the chromosome/scaffold represent 1 Mb. The colored bands linking chromosome/scaffold pairs represent syntenic blocks (minimum of 3 genes) shared by the species that are connected. The syntenic links are drawn with *T. gondii, H. hammondi* and *N.caninum* as the reference, in that order. Syntenic blocks were generated using genes present in orthologous clusters, where the cluster contained at least two genes, i.e. the gene is not present in all species. Data summarized in **Supplemental Table 2**.

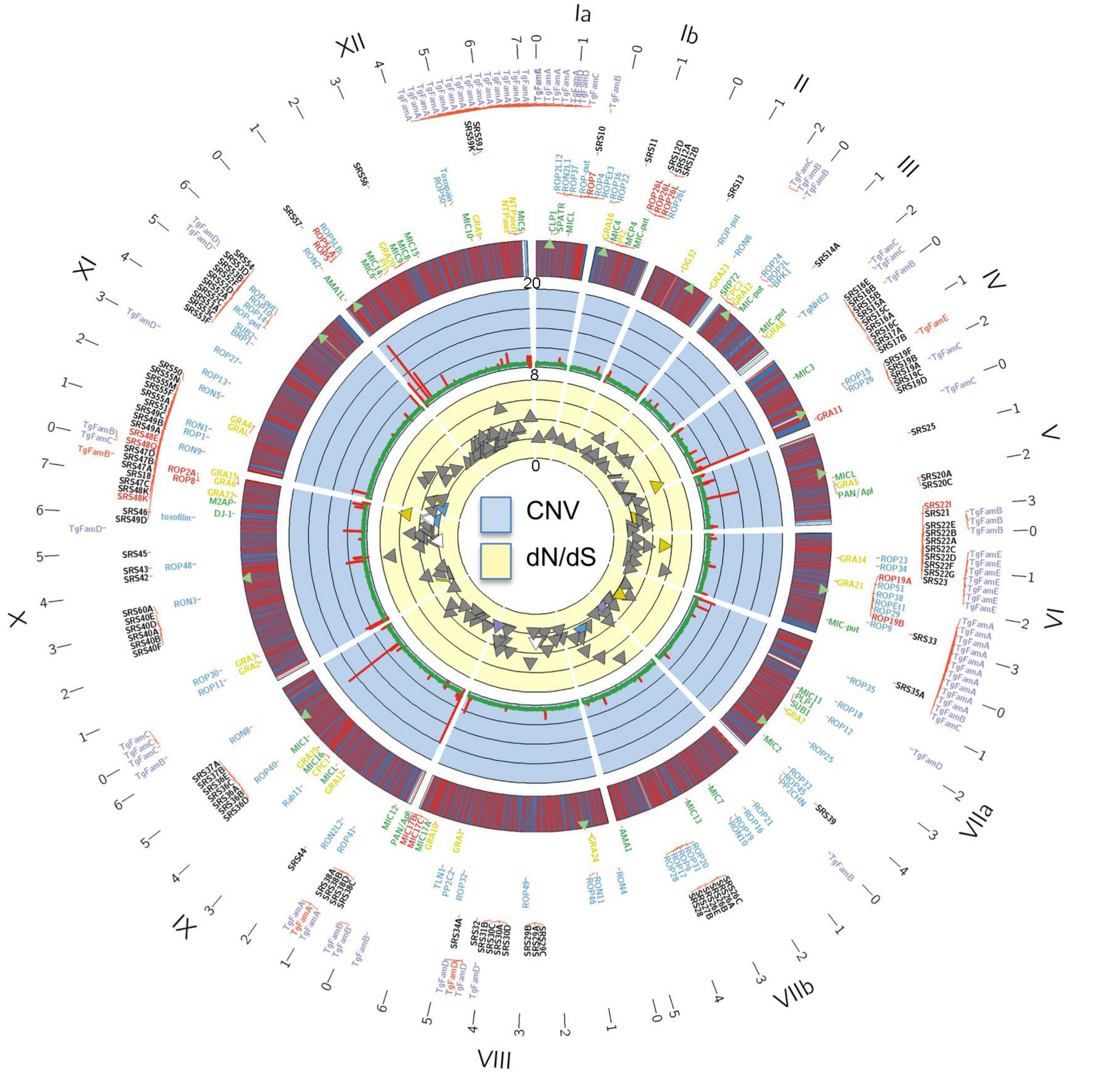

Supplemental Figure 4 Circos representational plot of Toxoplasma genome. The innermost yellow track is a scatter plot of genes with dN/dS ratio ≥ 2 (136 genes): y-axis 0 - 8, colors indicate SPD family. The track with blue background is a histogram of regions with CNV in ME49 using a rolling window of 2,500 bp: y-axis 0 - 20, 1X coverage (green), regions with CNV (red). The third circular track from the center plots the genes on 14 chromosomes: top strand gene (blue), bottom strand gene (red), centromere locations (green triangles). Outside of these circular tracks the locations of SPD genes are indicated in text. These categories ordered from the inside out are: *GRA* (yellow – 28 locations with 31 genes), *MIC* (green – 33 locations with 42 genes), *ROP* (blue – 71 locations with 104 genes), *SRS* (black – 47 locations with 115 genes), *TgFAM* (purple – 42 locations with 83 genes). In addition, genes with CNV are shown in red. Chromosome numbers (Roman) are shown on the outer ring, with size demarcations indicated by the Arabic numbers and tick marks.

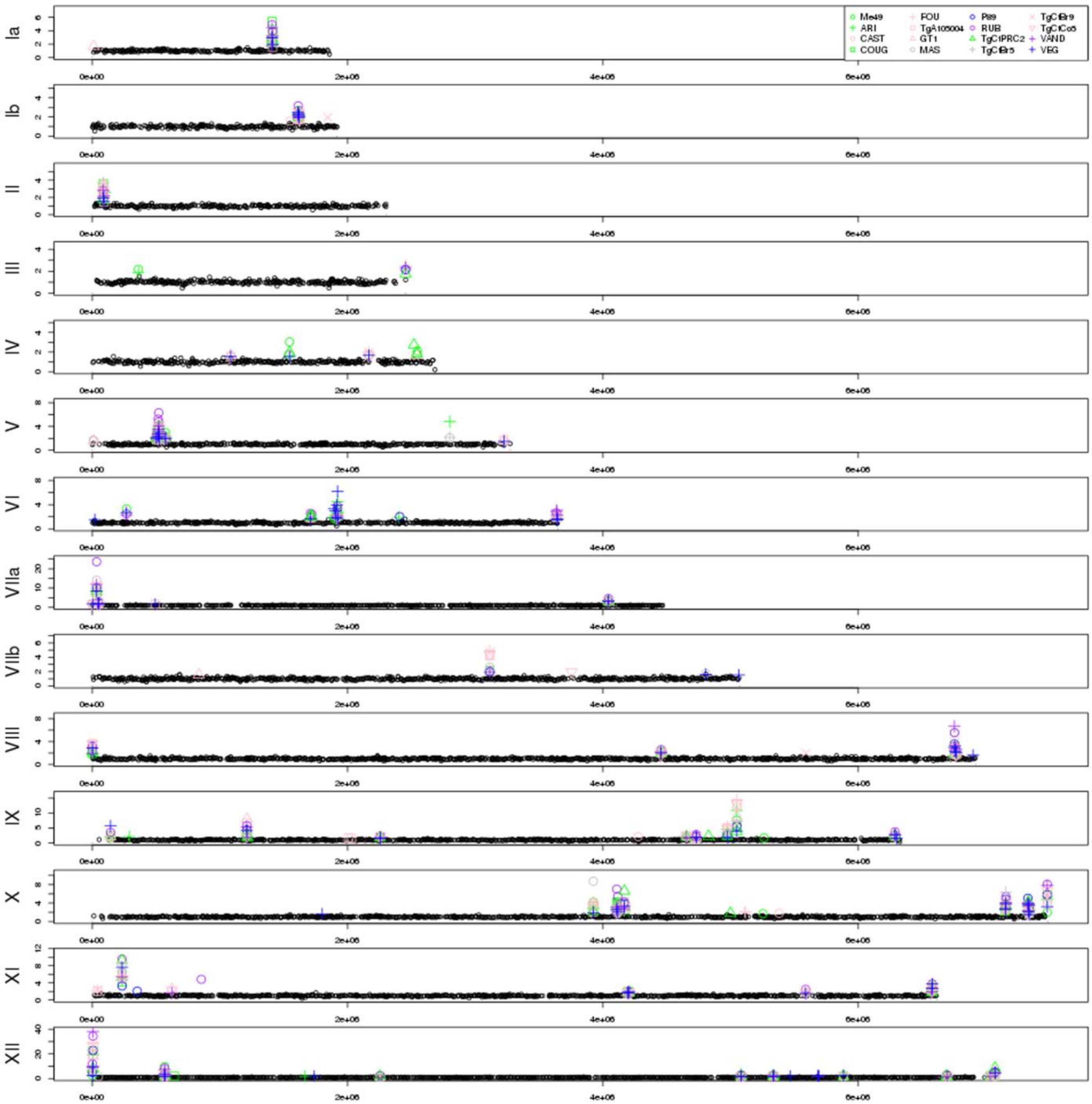

Supplemental Figure 5 Distribution of CNV in the 16 Toxoplasma reference genomes. Chromosome level plots of genes with CNV in the 16 Toxoplasma reference genomes. ME49 genes with no CNV (1X) are plotted by chromosomal location with black circles. Genes with CNV in each of the 16 reference genomes are plotted by chromosomal location with color based on Clade membership: Clade A (pink), Clade B (gray), Clade C (blue), Clade D (green), Clade E (yellow), Clade F (purple).

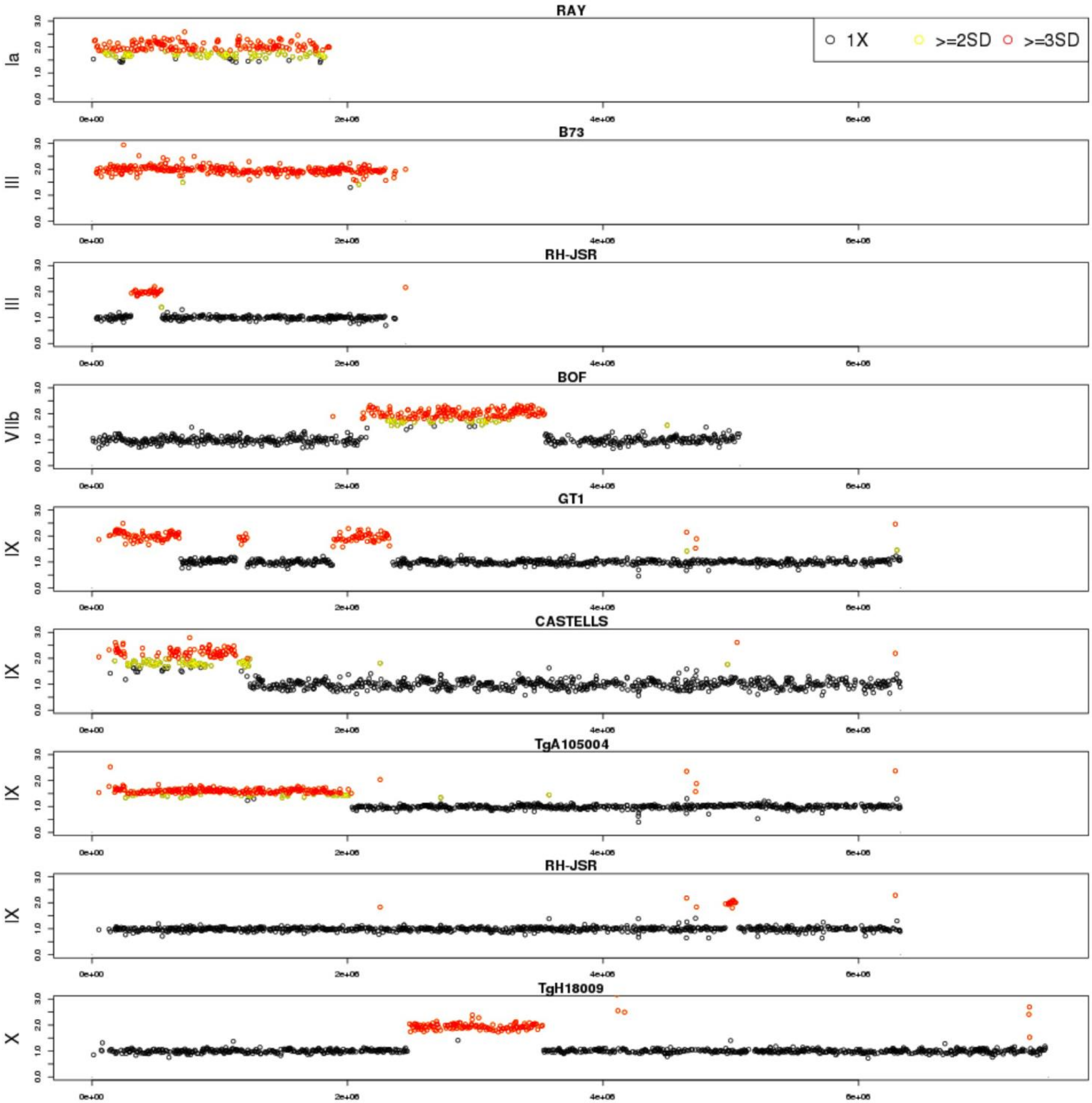

Supplemental Figure 6 Strains with aneuploidy or large duplicated regions. Select chromosomes are shown for Individual strains where 10 or more genes with CNV occur continuously. 1X genes (black), genes with > 2 X standard deviation (SD) CNV (yellow - borderline CNV), genes with > 3 X standard deviation (SD) CNV (red).

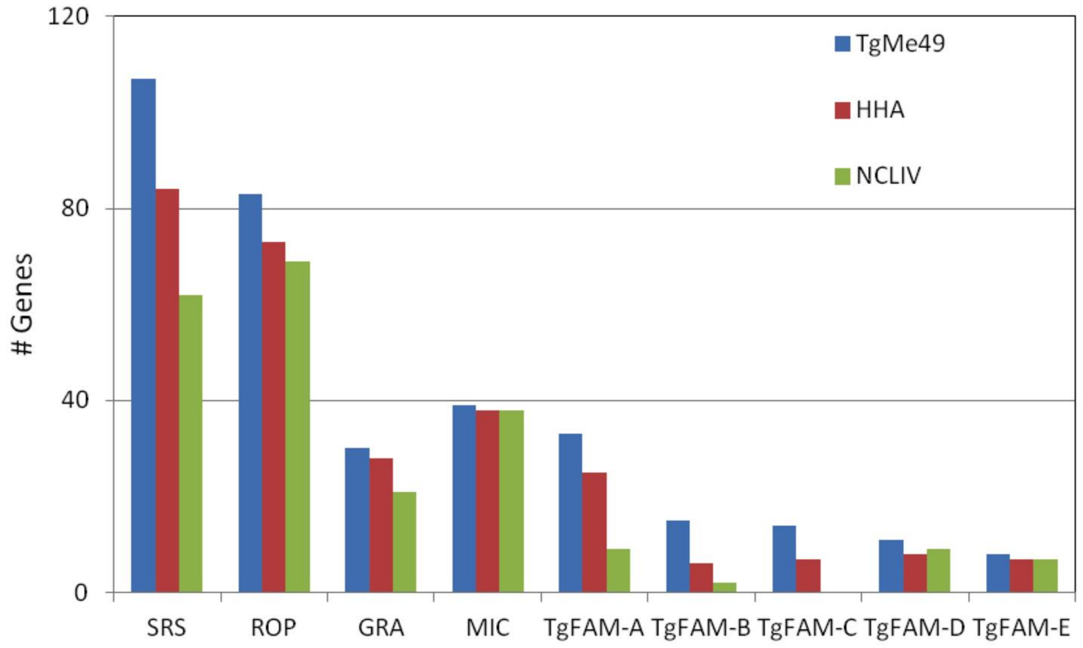

Supplemental Figure 7 Distribution of orthologous genes by species. Total number of genes for each category found in each of *T. gondii* (ME49 strain), *H. hammondi* (HHa) or *N. caninum* (NCLIV). Based on OrthoMCL clustering.

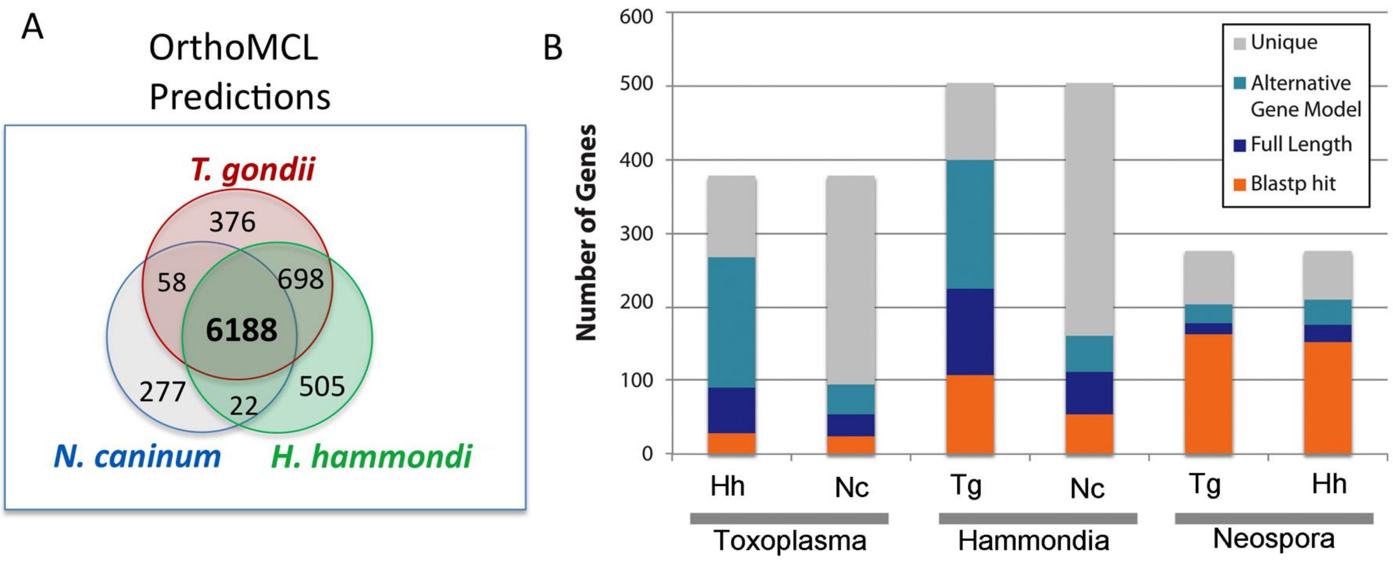

Supplemental Figure 8 Conserved genes among closely related tissue cyst forming coccidia. A) Number of shared and species-specific (unique) clusters of orthologous genes as defined by OrthoMCL. The number of clusters of orthologous genes shown for *T. gondii* represents those found in all 16 references strains. B) Analysis of species-specific genes as predicted by OrthoMCL. Orange bars (Blastp hit) represent species-specific genes that identify orthologs in the proteomes of the other two species with a blastp e-value ≤ $1x10^{-10}$ and 50% coverage of the shortest sequence. Blue bars (Full Length) depict species-specific genes that map as syntenic hits to the genomes of the other two species with at least 70% coverage using either GMAP or tblastn (e-value ≤ $1x10^{-5}$). These later two categories are likely distant orthologs that are classified as separate groups by OrthoMCL. Turquoise bars (Alternative Gene Model) represent the same as blue bars but for species-specific genes that map with less than 70% coverage or contain non-sense mutations or frame-shifts in the target genome. Gray bars (Unique) depict species-specific genes that do not match the proteomes or genomes of the other two species. Tg, *T. gondii*; Hh, *H. hammondi*; Nc, *N. caninum*.

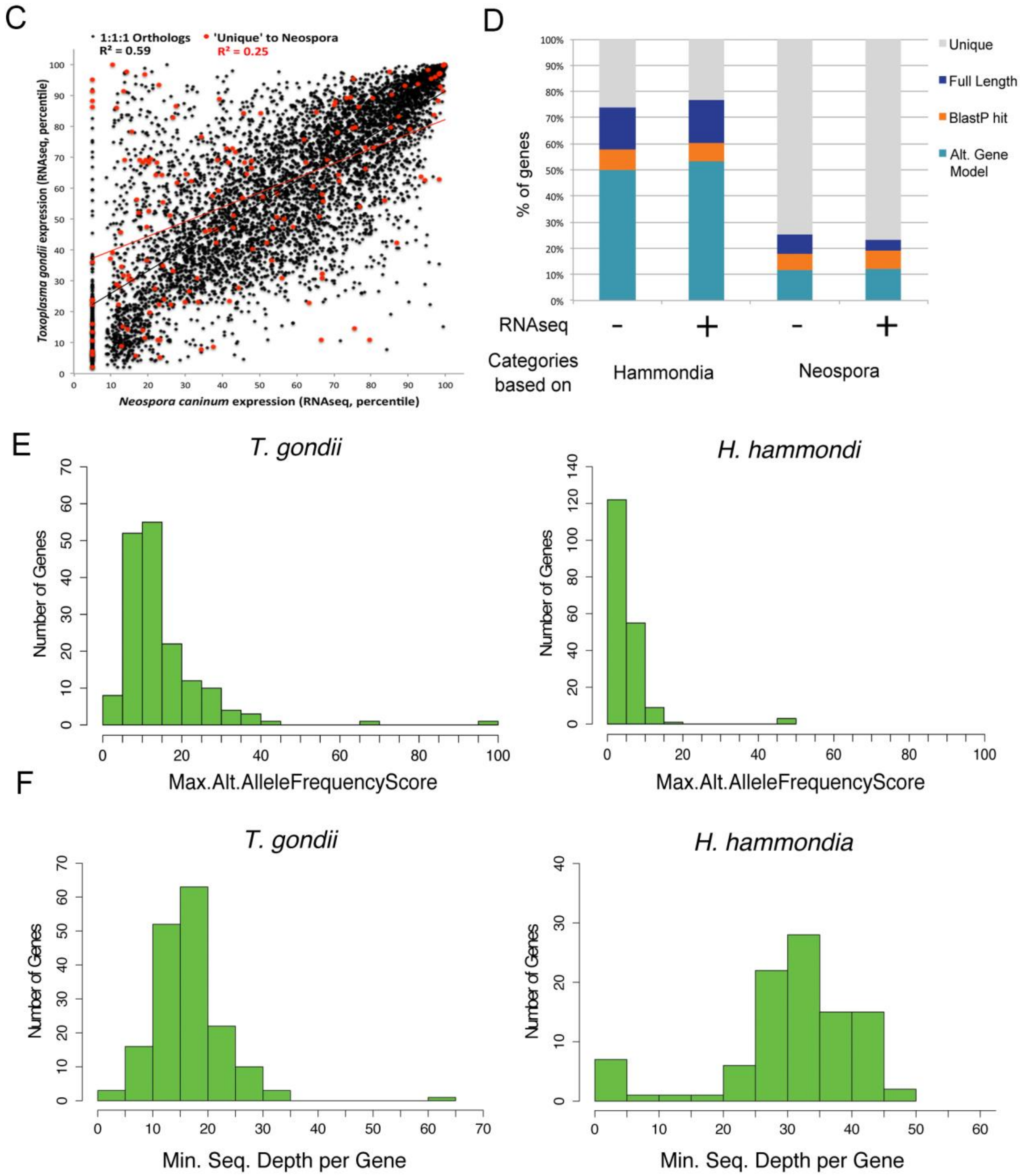

Supplemental Figure 8 Conserved genes among closely related tissue cyst forming coccidian continued.

C) Comparative gene expression for orthologous and predicted species-specific genes in *N. caninum* and *T. gondii*. For genes were there is a 1:1:1 orthologous correspondence between *N. caninum*, *T. gondii* and *H. hammondi* there was a good correlation of RNA-expression levels, based on RNA-seq data, between *N. caninum* and *T. gondii* (black dots). However for genes that were originally flagged as “unique” by OrthoMCL but for which there are annotated genes in both *N. caninum* and *T. gondii*,

the transcript levels did not correlate well (red dots). This suggests that these genes encode functionally different products and are not the result of assembly errors or inaccurate gene models.
D) RNA-seq analysis monitoring gene expression of putative species-specific genes in *T. gondii*. Plot shows the fraction of *T. gondii* genes from each of the four categories depicted in B that are supported (+) or not (-) by RNA-seq data. Potential *T. gondii* specific genes are defined by comparison to *H. hammondi* (left) and *N. caninum* (right). The expectation is that if unique and alternative gene models predicted in *T. gondii* were artifactual, they would be enriched in regions that lack RNA-seq data, compared to genes where there was evidence for orthologues being present (Full Length and BLASTP). Comparison of the genes in these different categories in regions with (+) and without (-) RNA-seq data showed no significant differences for any of the four gene categories ($P > 0.4$, Fisher's Exact Test). This result suggests that most unique genes and alternative gene models are not likely the result of artifactual gene predictions.
E) To assess if the high number of alternative gene models between *T. gondii* and *H. hammondi* was the product of genome sequencing errors we mapped genome sequencing reads to their respective *T. gondii* ME49 strain and *H. hammondi* genomes and calculated the maximum alternative allele frequency (MAAF) score within regions of putative species-specific genes. MAAF scores were defined as the maximum alternative allele frequency detected for SNPs and INDELs within a defined genomic region (see methods). It is expected that regions containing sequencing errors will present MAAF scores of ≥ 40%. This analysis revealed that only a small fraction (< 10 regions) of the loci containing potential alternative gene models in *T. gondii* (left) and *H. hammondi* (right) have MAAF scores ≥ 40%. This result suggests that frame-shifts and missense mutations found in alternative gene model loci are not the result of low sequencing quality in these regions.
F) Analysis of sequence coverage. More than 90% of the loci hit by alternative gene models in *T. gondii* and *H. hammondi* have a minimum sequencing depth higher than 5X. Left, *T. gondii* genomic regions similar to *H. hammondi* alternative gene model genes; right, *H. hammondi* genomic regions similar to *T. gondii* alternative gene model genes. These results also support the conclusion that alternative gene models are not due to poor sequencing quality.

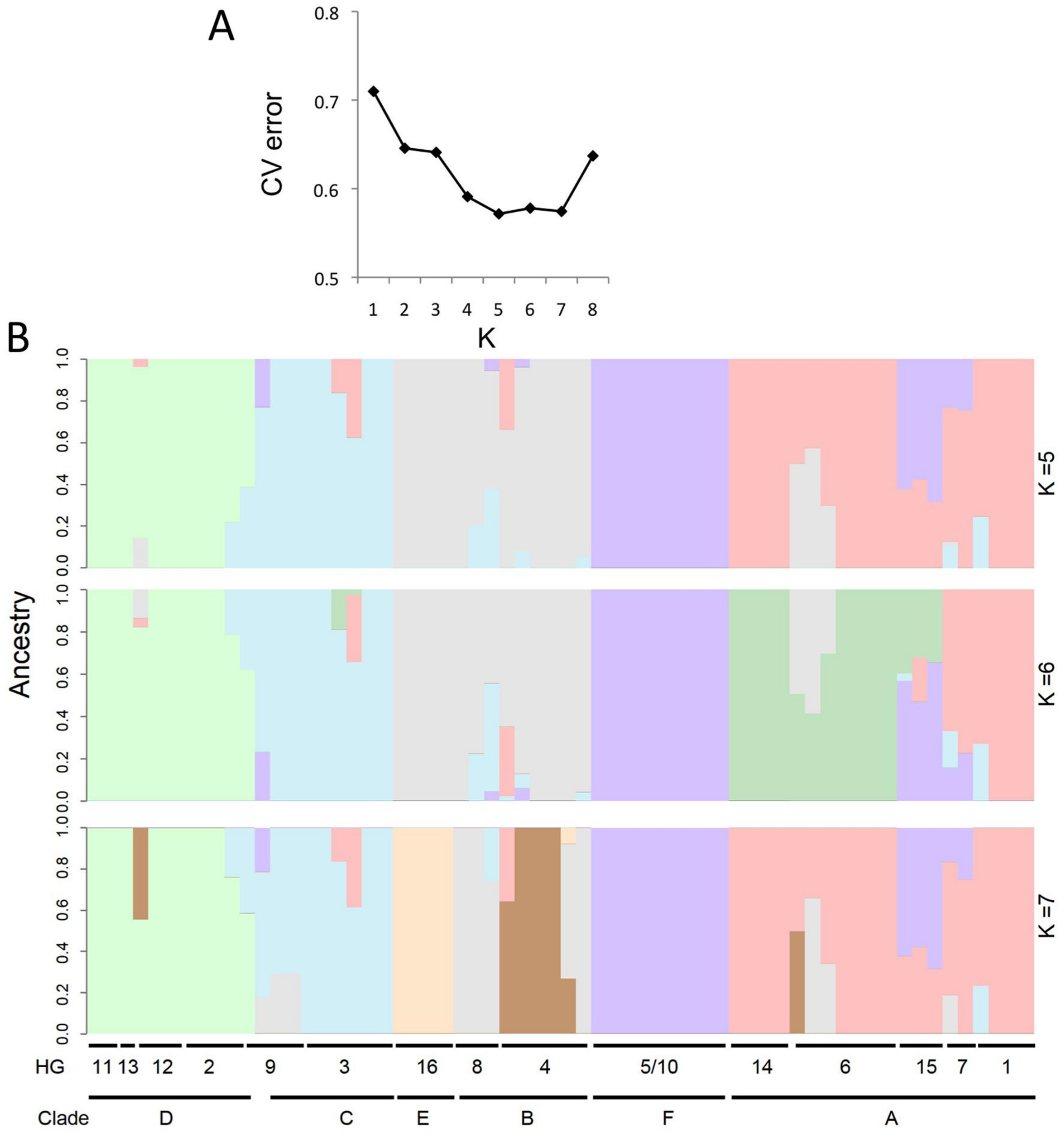

Supplemental Figure 9 Population structure inferred by Admixture analysis.
A) Cross validation (CV) plot was drawn for the whole genome SNPs data of *T. gondii*. Plot displays the CV error versus K values. CV errors for this dataset suggest K = 6 is the best fit. B) Admixture clustering analysis of *T. gondii* closely resembles the population clustering inferred by Neighbor-net analysis. Each individual in the plot is represented by a vertical stacked column of proportional genetic components of shared ancestry for K = 5 through K = 7. Each color represents each ancestral population. Previously designated haplogroups are indicated as HG.

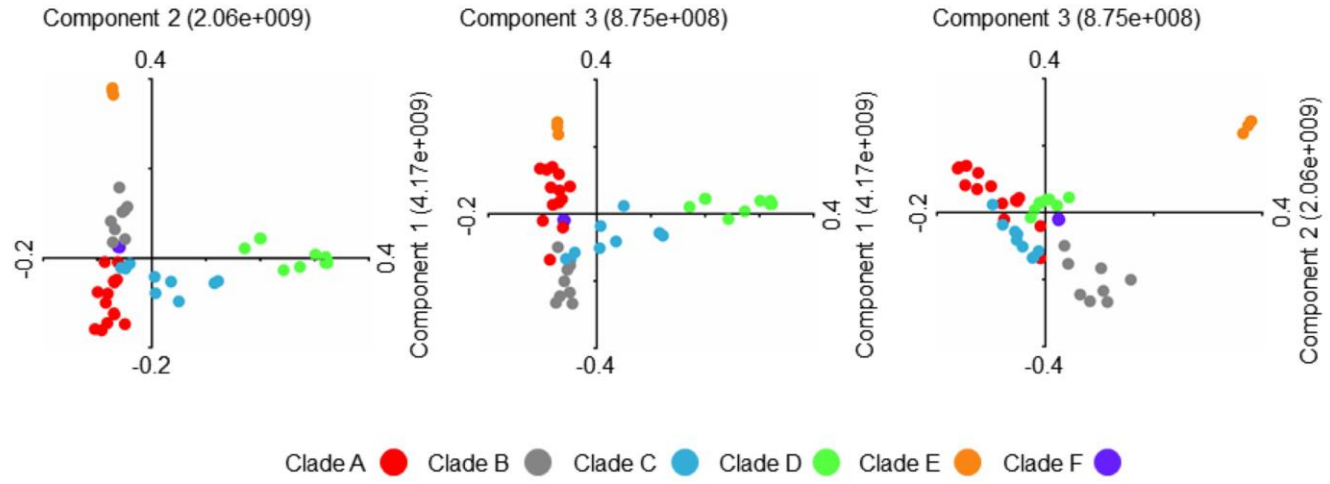

Supplemental Figure 10 Population genetic structure of *T. gondii* defined by principal component analysis. Color scheme of each clade of *T. gondii* follows **Fig. 3A**.

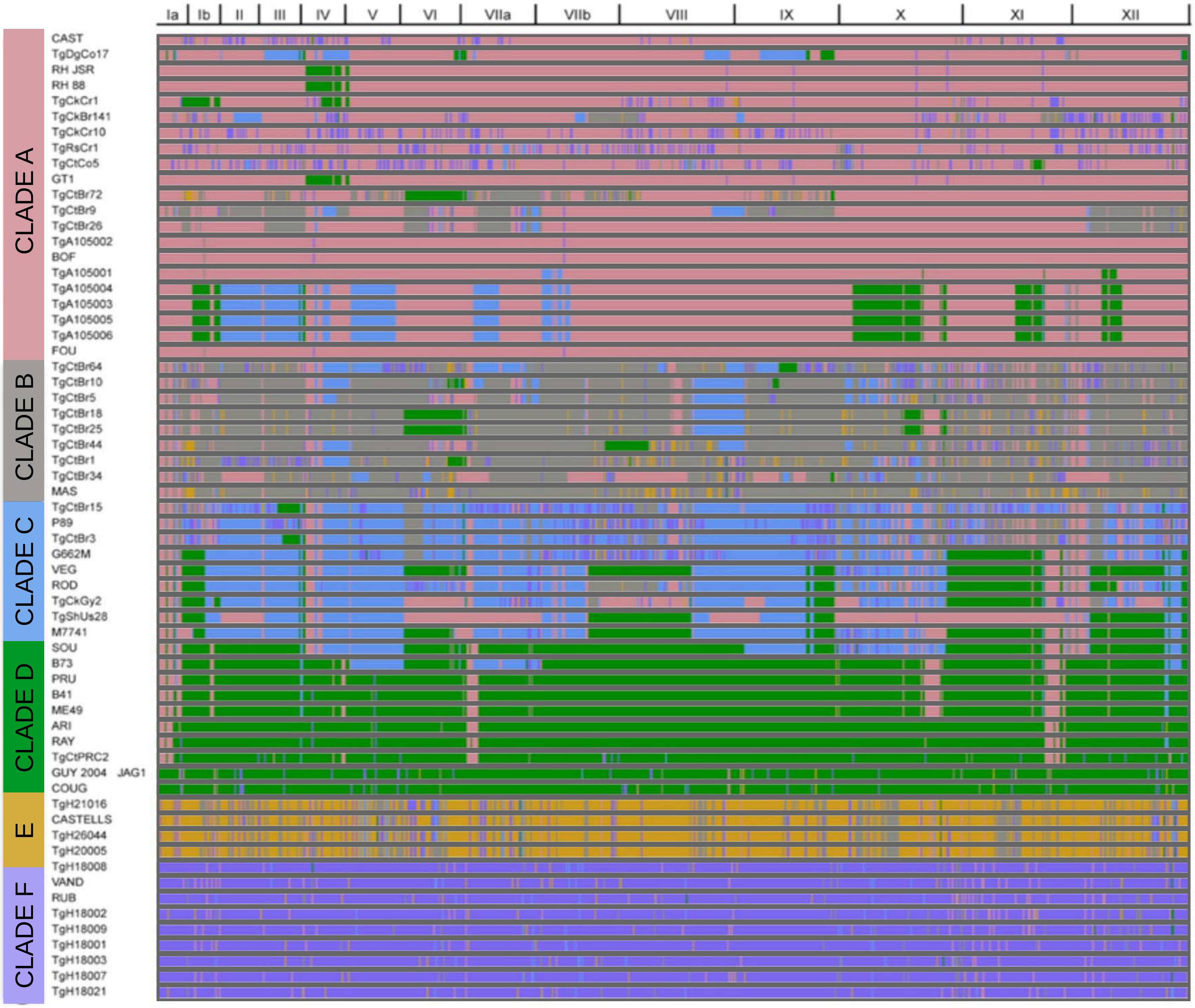

Supplemental Figure 11 Chromosome painting of 62 *Toxoplasma gondii* strains with local admixture analyses. Local admixture analyses were conducted on SNP blocks of size 1,000 bp on each of the 14 chromosomes. For each SNP block, local admixture assigned strains to a particular ancestral population. Sequences with the same color have high similarity, although this is not meant to imply origin. This analysis also revealed patterns of local admixture that suggest the occurrence of genetic crosses among strains of different clades, likely favored by their geographic proximity. For example, clade A strains TgCkBr141, TgCkCr10, TgRsCr1 and TgCtCo5 harbor a high number of small haploblocks that are shared with clade F strains (see purple bands for TgCkBr141, TgCkCr10, TgRsCr1 and TgCtCo5 strains and pink bands for clade F strains. Interestingly, all clade F strains were isolated from French Guiana while the aforementioned clade A strains were isolated from the nearby locations of Pará State, north of Brazil (TgCkBr141), Costa Rica (TgCkCr10 and TgRsCr1) and Colombia (TgCtCo5) (**Supplementary Data 1**). A similar high proportion of shared haploblocks occurs among several Brazilians strains from clades A (TgCtBr26, TgCtBr9 and TgCtBr72), B, and C (TgCtBr15, P89, and TgCtBr3)(see gray bands).

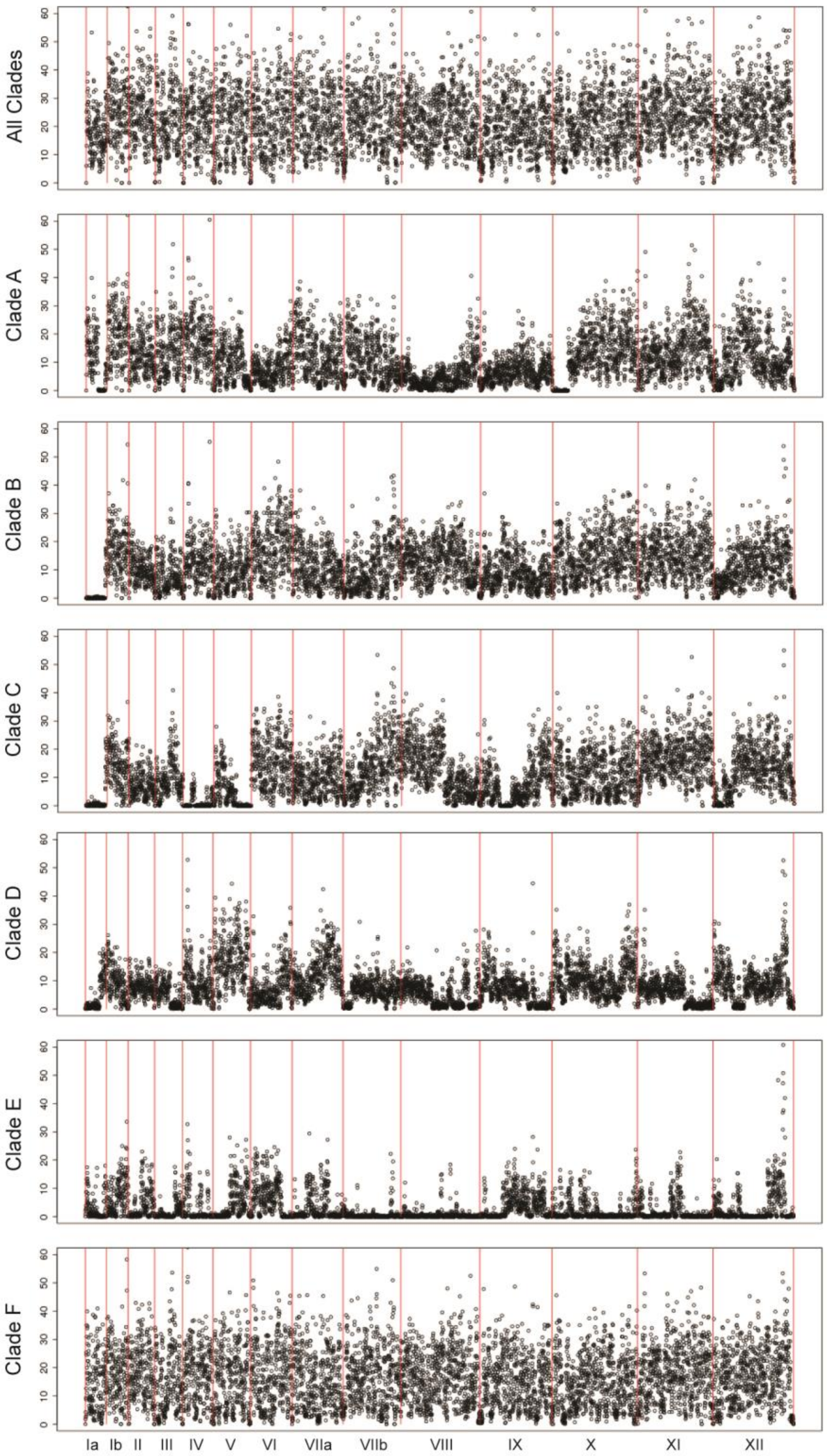

Supplemental Figure 12 Average SNPs per window comparing members of each clade. The average number of SNPs per 10 kb window for all strains within a Clade is plotted: y-axis 0 – 60 (outliers > 60 not plotted) red lines indicate chromosome boundaries. Large regions with low SNP rates for all members within a Clade are indicated by low regions in the plots. For example, chromosome Ia has low SNP rates when comparing all members of Clade B, but not for Clade F. Chromosome numbers are indicated along the x-axis.

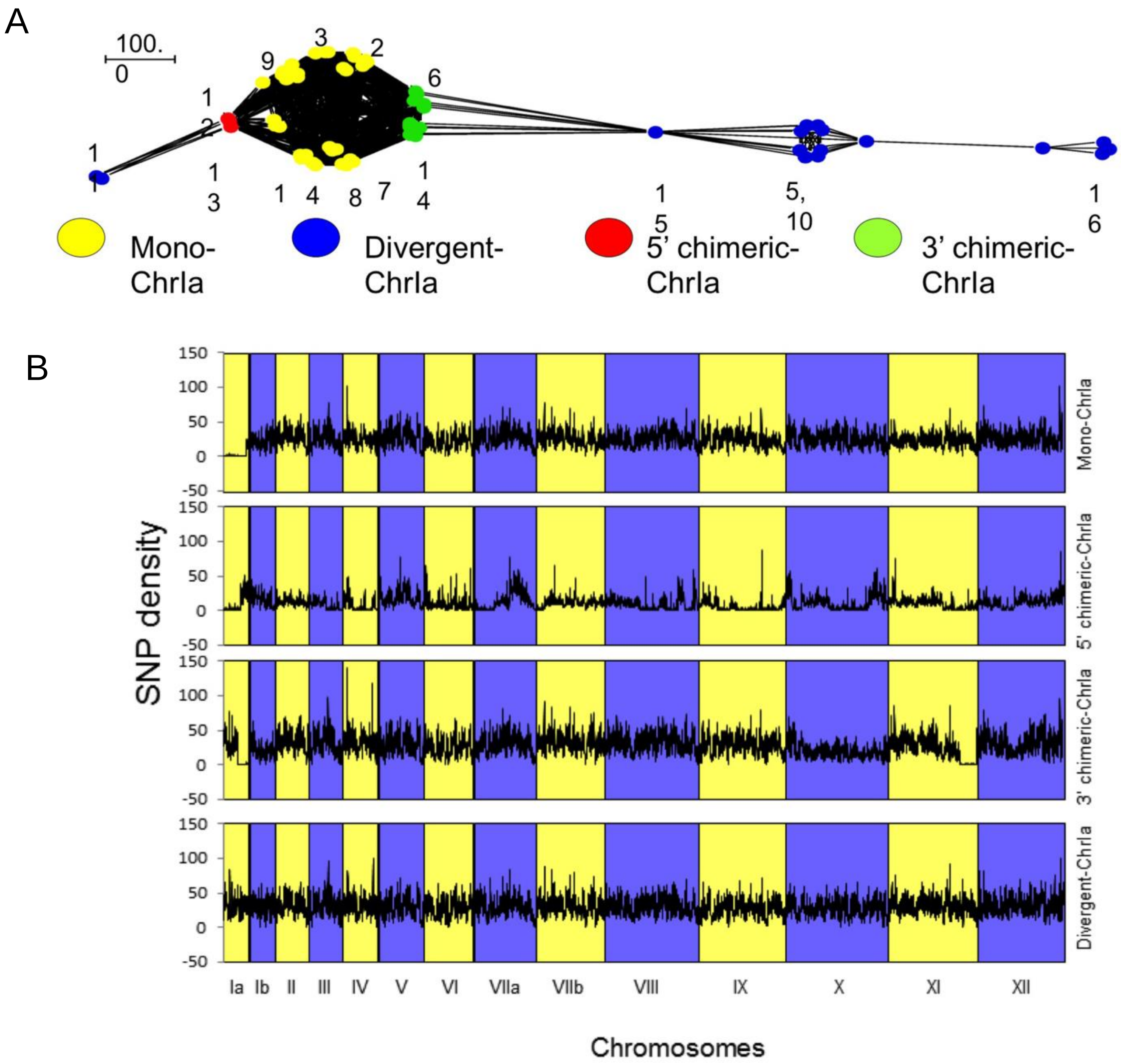

Supplemental Figure 13 Expansion of monomorphic version of ChrIa among *T. gondii* isolates.

A) Minimum spanning network analysis of ChrIa shows four major clusters; mono-ChrIa, divergent ChrIa, 5' chimeric-ChrIa, and 3' chimeric-ChrIa. B) SNP density plots indicate the monophyletic distribution of mono-ChrIa among the majority of *T. gondii* strains. Strains used in each of these plots were defined by minimum spanning network clustering of ChrIa (A). X-axis indicates the relative physical distance of 14 chromosomes of *T. gondii*: y-axis indicated the number of SNPs per 10 kb sliding window.

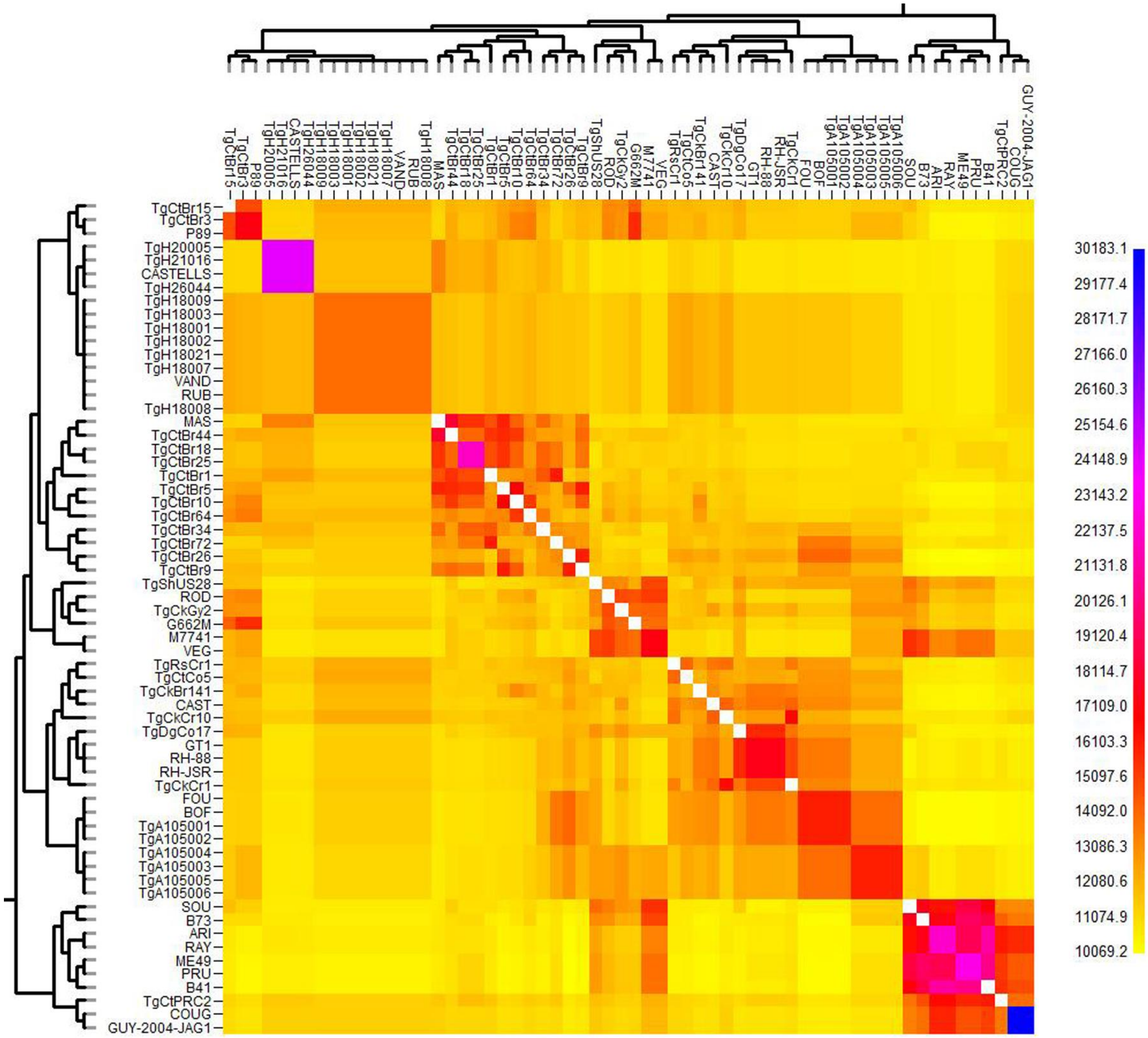

Supplemental Figure 14 Population structure of *T. gondii* inferred by Bayesian clustering. Pair-wise co-ancestry heatmap reveals the shared haplotypic segments (“chunks”) calculated based on genome wide SNP data of *T. gondii*.

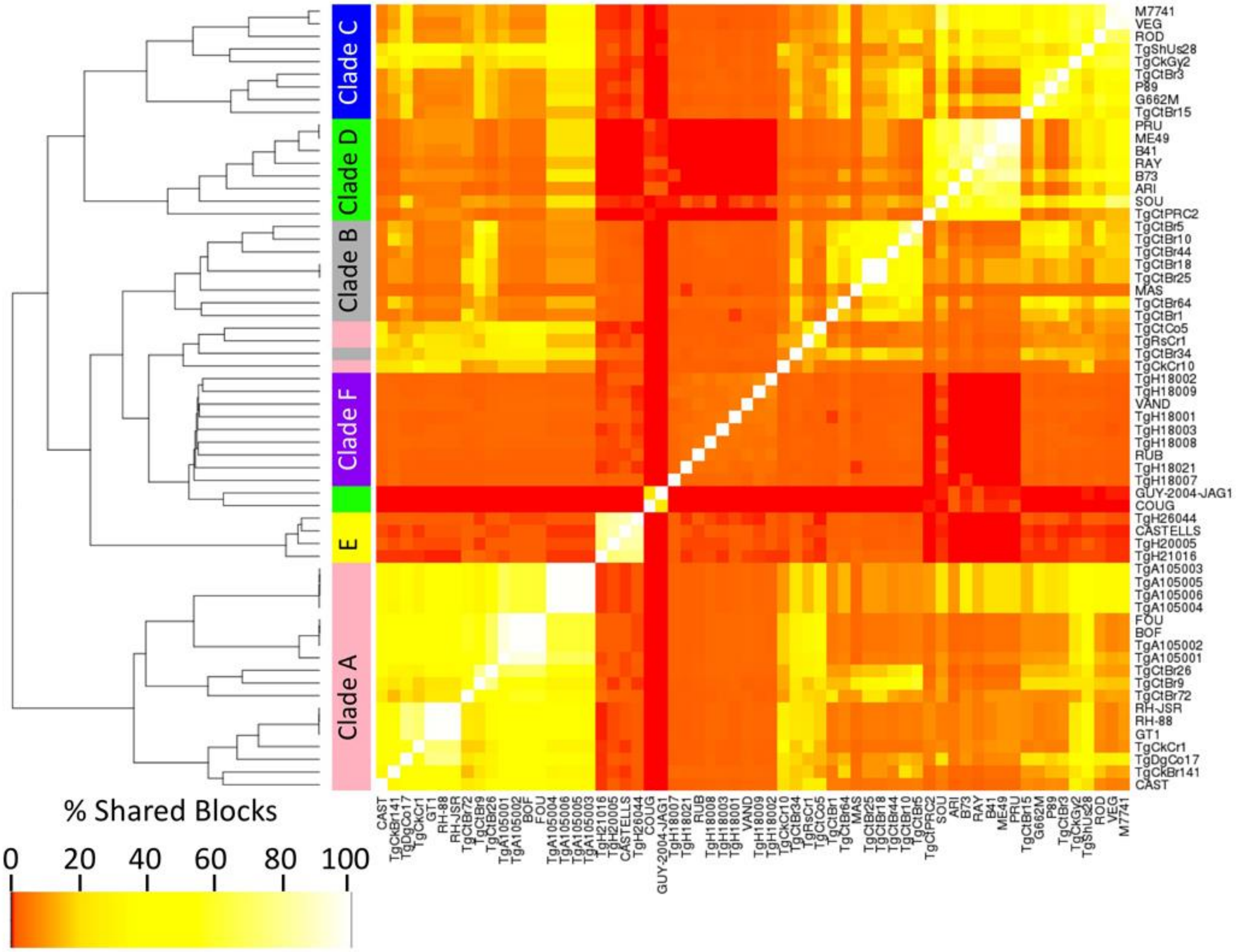

Supplemental Figure 15 Heatmap clustering of co-inheritance of shared blocks. The percentage of shared blocks between two strains was determined for all 62x62 pair-wise strain comparisons (1953 non-redundant comparisons): scale = % shared blocks. Hierarchical clustering on percent shared blocks independently grouped the strains by Clade.

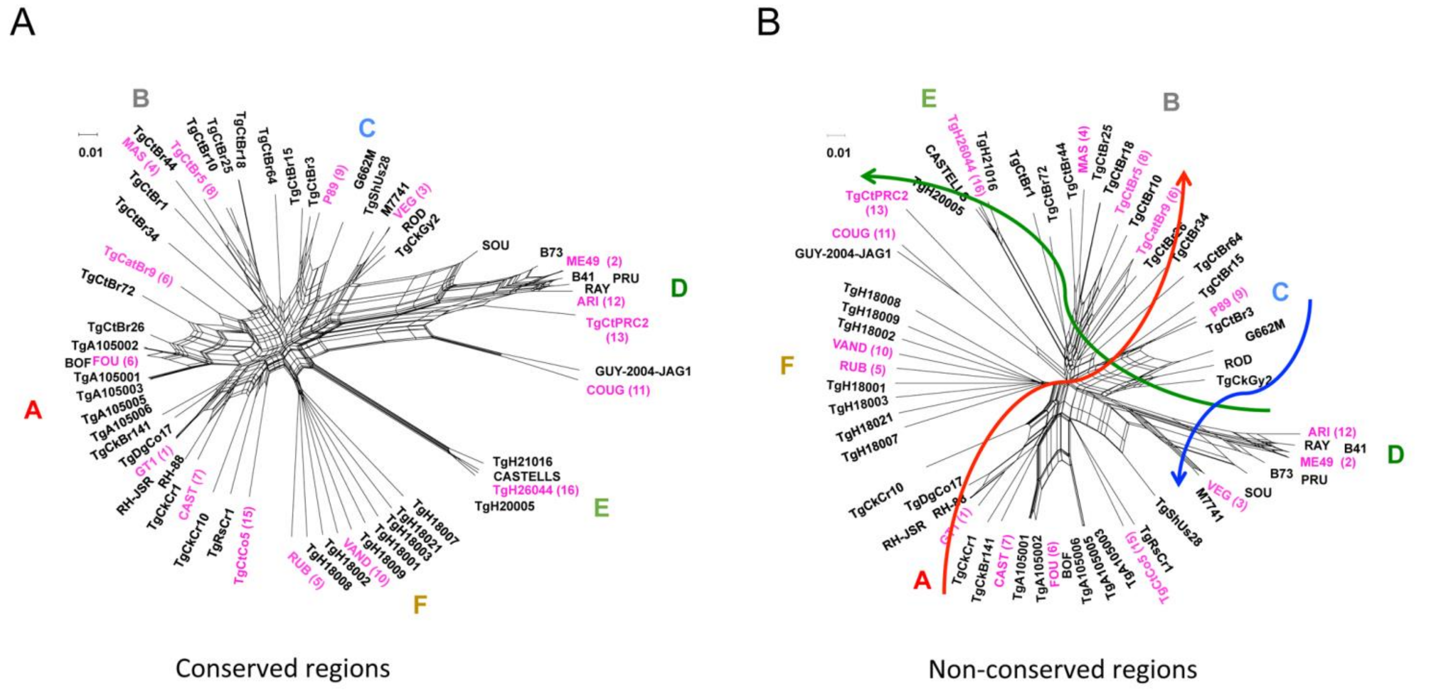

Supplemental Figure 16 Neighbor-net trees for conserved (A) vs. non-conserved (B) regions of the genome based on total SNPs for the 62 strains. Reference strains for haplogroups (numbers indicated in parentheses) are show in pink. A number of strains group differently in the non-conserved network compared to the conserved network: stains VEG, M7741, and TgShUs28, which are part of clade C, realigned to clade D (blue arrow). Strains TgCtBr9 (6) and TgCtBr72, which are part of clade A, regroup to form part of clade B (red arrow). Finally, strains TgCtPRC2 (1), COUG (11) and GUY-2004-JAG1, which are part of clade D, regroup to a separate long branch (green arrow). Networks were generated as described in Figure 4a. Scale = number of SNPs per site.

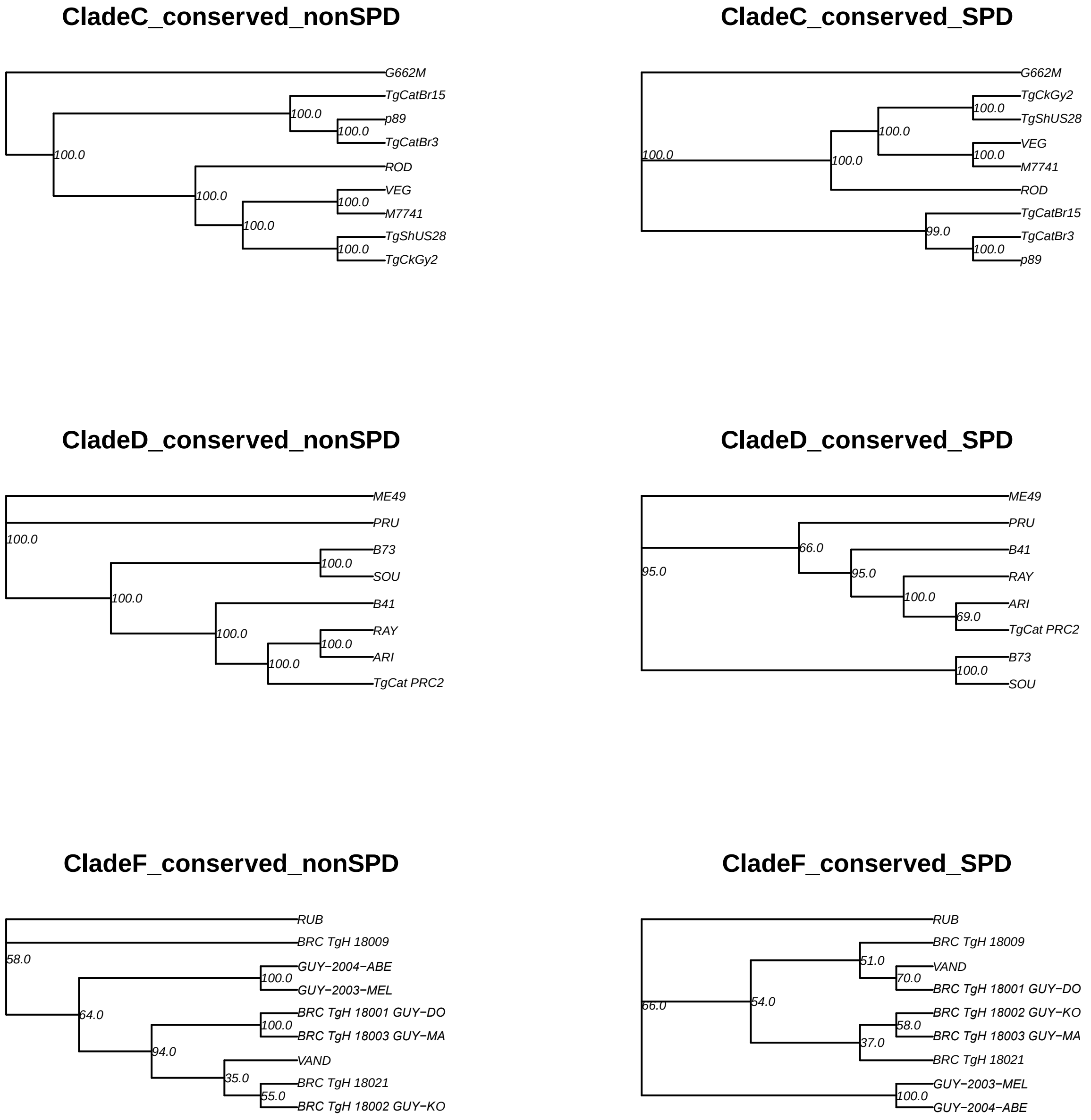

Supplemental Figure 17 Phylogenetic trees for SPD and non-SPD genes within conserved regions for Clades C, D, and F. Trees were generated with 100 bootstraps and support values are shown at the branches**.** Trees generated from SPD genes closely resemble those of the non-SPD genes, reflecting their common, recent shared ancestry. Trees were generated using RMxML with the GTR+GAMMA model as further described in the methods.

Supplementary Table 1 Genome assemblies for *T. gondii* reference strains.

	T. gondii GT1	T. gondii VEG	T. gondii ME49	T. gondii MAS	T. gondii RUB	T. gondii CAST	T. gondii TgCtBr5	T. gondii P89	T. gondii VAND	T. gondii COUG	T. gondii ARI	T. gondii TgCtPRC2	T. gondii GAB2-2007-GAL-DOM2	T. gondii TgCtCo5	T. gondii FOU	T. gondii TgCtBr9	H. hammondi H.H.34
Assembly size	64.1 Mbp	63.7 Mbp	65.5 Mbp	61.7 Mbp	62.8 Mbp	63.3 Mbp	62.1 Mbp	62.0 Mbp	62.6 Mbp	64.9 Mbp	63.3 Mbp	63.0 Mbp	63.7 Mbp	63.0 Mb	62.6 Mbp	62.0 Mbp	67.4 Mbp
Number of contings (bp)	3,364	3,424	2,532	6,767	7,495	7,292	9,658	6,415	5,780	15,676	7,609	8,277	7,812	8,277	10,363	7,392	16,398
Contig N50 (bp)	351,608	368,606	1,219,553	38,798	30,445	46,384	26,933	41,694	61,086	33,690	40,798	35,133	59,599	35,133	33,296	35,035	84,429
Number of scaffolds (bp)	313	306	2,266	201	165	127	212	170	2,137	158	126	143	149	143	216	165	14,861
Scaffolds N50 (bp)	4,191,897	3,747,630	6,301,488	1,390,389	2,283,863	3,297,333	1,365,820	1,730,166	1,742,129	3,382,402	2,667,080	1,948,981	2,099,269	1,948,981	1,635,587	1,489,074	1,494,935
Number of fragment 454 reads	NA	NA	1,240,227	NA	1,559,686	NA	NA	NA	2,907,070	NA	NA	NA	NA	2,680,081	NA	NA	3,144,203
Number of 3kbp 454 reads	NA	NA	3,065,409	5,242,673	5,185,312	4,259,026	5,734,612	5,857,993	3,803,275	2,012,690	2,294,377	3,693,418	8,773,773	3,860,620	6,228,112	6,307,240	1,330,431
Number of 8kbp 454 reads	NA	NA	1,182,346	2,362,125	3,111,736	2,856,093	2,233,469	3,038,593	2,465,760	887,134	1,232,621	NA	NA	2,543,092	3,455,458	1,847,984	NA
Number of Illumina PE reads	94,318,960	99,517,042	NA	48,698,504	11,679,625	84,807,782	34,799,463	17,351,409	29,959,353	29,851,932	24,762,190	30,235,296	111,457,054	61,748,554	24,768,278	16,761,892	60,610,959
Number of Sanger reads	1,415,530	1,352,018	980,812	NA	NA	NA	NA	NA	NA	NA	NA	NA	NA	NA	NA	NA	NA
Average sequencing depth	67.44x	77.38x	26.55x	42.75x	30.56x	74.24x	31.5x	26.85x	39x	45.9x	46.2x	28.52x	98.92x	53.3x	31.05x	24.5x	66.13x
Accession Number	GCA_000149715.2	GCA_000150015.2	GCA_000006565.2	GCA_000224865.2	GCA_000224805.2	GCA_000256705.1	GCA_000259835.1	GCA_000224885.2	GCA_000224845.2	GCA_000338675.1	GCA_000250965.1	GCA_000256725.1	GCA_000325525.2	GCA_000278365.1	GCA_000224905.2	GCA_000224825.1	GCA_000258005.2

Supplementary Table 2 Synteny Data

Levels of synteny based on orthologous genes (2067 clusters) present in all species

	TG vs HH	TG vs NC	TG vs SN	TG vs ET	HH vs NC	HH vs SN	HH vs ET	NC vs SN	SN vs ET
**Total number of syntenic blocks**	16	30	95	0	28	95	0	93	0
**Total number of genes in syntenic blocks**	2115	2064	853	0	2062	862	0	836	0
**Number of Mb in syntenic blocks for species 1**	59.88	58.67	19.21	0	58.47	19.2	0	18.16	0
**Number of Mb in syntenic blocks for species 2**	59.49	55.51	35.38	0	55.55	35.82	0	34.72	0
**Percent proteome in syntenic blocks for species 1**	23.71	23.14	9.56	0	26.14	10.93	0	11.51	0
**Percent proteome in syntenic blocks for species 2**	26.81	28.41	12.07	0	28.38	12.2	0	11.83	0

Levels of synteny based on orthologous genes (7391 clusters) present in at least two species

	TG vs HH	TG vs NC	TG vs SN	TG vs ET	HH vs NC	HH vs SN	HH vs ET	NC vs SN	SN vs ET
**Total number of syntenic blocks**	16	46	159	0	44	155	0	145	0
**Total number of genes in syntenic blocks**	7090	6307	1678	0	6294	1659	0	1525	0
**Number of Mb in syntenic blocks for species 1**	60.79	62.24	28.42	0	61.83	28.13	0	25.19	0
**Number of Mb in syntenic blocks for species 2**	60.34	58.75	53.72	0	58.68	54.04	0	50.33	0
**Percent proteome in syntenic blocks for species 1**	79.48	70.71	18.81	0	79.78	21.03	0	20.99	0
**Percent proteome in syntenic blocks for species 2**	89.87	86.8	23.75	0	86.62	23.48	0	21.58	0

Materials and Methods

Propagation of strains and isolation of gDNA

Sixty two representative strains of *T. gondii* were selected from different haplogroups from around the world (Dataset 1) [1]. Strains were cultured in human foreskin fibroblast (HFF) cells maintained in DMEM (Invitrogen) containing 10% FBS, 2 mM glutamine, 20 mM HEPES pH 7.5 and 10 µg/ml gentamicin, and harvested after host cell lysis by passing through 3.0 micron polycarbonate filters (Fisher Scientific, UK). Harvested parasites were resuspended in phosphate buffered saline (PBS) at a concentration of approximately 10^7 cell/ml and genomic DNAs were prepared using DNeasy Blood and Tissue kit (Qiagen, USA) according to the manufacturer's instructions.

Genome sequencing and assembly of *T. gondii* reference strains

Improvement of the existing *T. gondii* ME49 assembly was carried out using a combination of Sanger and 454 sequencing technologies. Briefly, one fragment and three paired-end (PE) 454 libraries with 3 kbp (done in duplicate) and 8 kbp inserts were prepared from total genomic DNA (gDNA) extracted from tachyzoite cultures and sequenced in four full plates of a 454 Titanium FLX sequencer. The resulting 5.4 million reads were combined with 980,812 Sanger PE reads from 3 kbp, 10 kbp, and 15 kbp insert libraries generated for the existing genome sequence, screened for contamination and reassembled using Celera Assembler software [2]. Scaffolds were then aligned with MUMmer [3] to *T. gondii* ME49 chromosome sequences from ToxoDB v8.0 to generate super-scaffolds spanning entire chromosomes. The final *T. gondii* ME49 assembly (GenBank ABPA00000000.2, ToxoDB v9.0) is 65.9 Mbp long with an average read depth of 26x and a contig N50 of 1.2 Mbp (Supplementary Table 1).

To reassemble the genome sequences from *T. gondii* GT1 and VEG strains, Illumina PE sequencing libraries with an average insert size of 300 bp were prepared from total gDNA extracted from tachyzoites cultures from each strain and sequenced in one seventh of an Illumina HiSeq 2000 lane. Thereafter, Illumina PE reads (42.5 M for GT1 and 89.7 M for VEG) were examined for contamination, pooled with Sanger PE reads from 3 kbp and 10 kbp libraries (707,774 for GT1 and 676,028 for VEG) from their respective genome assemblies and assembled with Newbler v2.6 [4]. Resulting GT1 and VEG assemblies were deposited in GenBank with accession numbers AAQM00000000.3 and AAYL00000000.2, respectively. Genome sequencing of the remaining reference *T. gondii* strains (Supplementary Table 1) was performed using a 454-Illumina hybrid approach. For each strain up to three 454 gDNA sequencing libraries (one fragment, one 3 kbp PE

and one 8 kbp PE libraries) and one Illumina PE library (300 bp inserts) were prepared from gDNA extracted from tachyzoite cultures. Each library was sequenced using either one (fragment or 8 kbp libraries) or two (3 kbp library) full plates of a 454 Titanium FLX run or one half of an Illumina GAII lane. After removing contaminating sequences Illumina and 454 reads were assembled with Newbler v2.6 and the final assembly was submitted to GenBank. Supplementary Table 1 provides assembly statistics as well as GenBank accession numbers.

Genome sequencing of non-reference *T. gondii* strains for SNP discovery

For each non-reference strain, a single Illumina PE barcoded library was prepared from tachyzoite gDNA. Libraries were then pooled into groups of nine samples and sequenced multiplexed in a single lane of an Illumina HiSeq 2000 machine. Sequencing reads were deposited in the Sequence Read Archive repository (SRA) at NCBI (Supplementary Dataset 1).

Sequencing of tachyzoite mRNA samples

To aid in the curation of ME49 gene models, two tachyzoite-specific Illumina cDNA libraries were constructed from mRNA isolated from tachyzoite cultures from ME49 and GT1 strains. Each library was then sequenced in a single lane of an Illumina Genome Analyzer II machine. Reads were deposited in SRA with accession numbers SRR350746 (ME49) and SRR516419 (GT1).

Structural and functional annotation of the *T. gondii* ME49 genome

Protein sequences from GenBank NR and Pfam-seed databases [5] as well as from the ME49 proteome were aligned to assembled sequences with the utility NAP from the AAT-package [6] to generate protein-based evidence to support identification of gene structures. To train ab-initio gene finders, a tachyzoite-specific RNAseq dataset [7] was obtained from ToxoDB, assembled with Trinity [8] and aligned to the new ME49 assembly sequence with PASA [9]. In addition, a second dataset composed of 136,229 tachyzoite-specific *T. gondii* cDNA/EST Sanger sequences were downloaded from GenBank and then assembled and aligned to the new genome assembly with PASA. This collection of aligned transcripts was used to generate a training set of ~400 manually curated genes, supported by full-length transcripts. GlimmerHMM [10], GeneZilla [10] and Augustus v2.5 [11] were trained and run on the ME49 scaffolds to predict potential protein-coding gene structures. Augustus was run a second time using aligned transcripts as hints to increase prediction accuracy.

The 7,987 gene annotations from the previous assembly were mapped as predictions onto the new genome sequence at three different stringencies with three different sets of programs: (i) MUMmer (nucleotide-based mapping, high stringency), (ii) PASA (nucleotide-based mapping,

medium stringency), and (iii) GenWise [12] followed by Geneid [13] (protein-to-nucleotide-based alignment, low stringency). Only three genes, encoding for hypothetical proteins, did not align with any of these methods and hence were discarded. Once all evidence was mapped to the new genome sequence a first set of preliminary working genes models was generated with EVidenceModeler [14] (EVM), a program that predicts gene structures as a weighted consensus of all the evidence available at each particular locus, including transcript and protein alignments and gene predictions. Further improvement of gene structures was performed with PASA by the incorporation of additional transcriptomic evidence from an oocyst-specifc (from the type 2 M4 strain, http://tinyurl.com/mdkdbup) [15] and a bradyzoite-specific (from the type 2 ME49 strain, http://tinyurl.com/ons4zdo) Illumina RNAseq datasets downloaded from ToxoDB. Briefly, each RNAseq dataset was assembled with Trinity and transcripts aligned to the ME49 assembly sequence with PASA. Thereafter, a second functionality of PASA was used to compare and detect annotation inconsistencies between working genes models and mapped transcripts, such as genes that should be created, merged, split or incorporate new exons. A final round of manual curation was performed by the incorporation of structural changes deposited by the scientific community in ToxoDB.

Functional annotation of ME49 protein-coding genes

Predicted peptides from ME49 working models were run through JCVI's autonaming pipeline, that assign product names based on a number of sequence similarity searches whose results are ranked according to a priority rules hierarchy. These analyses included Blastp searches against the previous *T. gondii* ME49 proteome and the GenBank non-redundant protein database, HMM searches against Pfam and TIGRfam [16] databases, and RPS-Blast searches against the NCBI-CDD database [17]. Proteins without any significant hit to other proteins or protein domains were flagged as "hypothetical protein". The final list of product names was then curated by researchers from the Toxoplasma research community before being assigned to working models.

To predict the possible subcellular localization of predicted peptides, potential signal peptides and transmembrane domains were predicted with signalP and TMHMM programs respectively [18]. Enzyme Commission (EC) numbers were assigned with PRIAM [19] and curated based on annotations deposited by the scientific community in ToxoDB and those kindly provided by Dr. John Parkinson. Gene ontologies were annotated based on pfam2go and ec2go mappings, annotations from the ApiLoc database (http://apiloc.biochem.unimelb.edu.au) and those curated by the research community in ToxoDB.

Assignment of gene pub_locus identifiers

ME49 protein-coding genes from the previous genome assembly inherited a modified version of their pub_locus identifiers by the addition of 100,000 to their number suffixes (for example, pub_locus TGME49_012345 became TGME49_112345 in the new assembly). Annotated tRNAs, rRNAs and newly predicted protein-coding genes were assigned completely new pub_locus identifiers. For the annotation of the remaining *T. gondii* reference genomes and *H. hammondi*, protein-coding genes that could be mapped from the newest ME49 annotation inherited their pub_locus suffix numbers while the rest of the genes acquired new pub_locus identifiers. Partial genes that based on the evidence were split into two or more fragments on different contigs kept the same pub_locus suffix number followed by a letter, different for each fragment.

Domain Identification and characterization of *T. gondii* novel gene families

To identify known protein domains the *T. gondii* ME49 proteome was searched against Pfam and TIGRfam HMM profiles using HMMER3 [20]. Any protein segment scoring above the trusted cutoff assigned to a particular HMM profile was assigned to that domain. Known domain sequences were then removed from protein sequences and remaining peptides searched against each other using Blastp to identify potential novel domains not represented in Pfam and TIGRfam databases. Similar peptide sequences were clustered by creating a link between any two protein sequences having an identity above 30% over an span of at least 50 residues and an e-value < 0.001. The Jaccard coefficient of community [21] was estimated for each linked pair of peptide sequences *a* and *b* as follows:

$$J_{a,b} = \frac{\#\text{ distinct accessions matching } a \text{ and } b \text{ including } (a,b)}{\#\text{ distinct accessions matching either } a \text{ or } b}$$

with the Jaccard coefficient (Ja,b), named link score, providing a measure of similarity between the two proteins. Associations between peptides that had an insufficient link score were eliminated, and the remaining links were used to generate single linkage clusters. Clustered peptides were then aligned using ClustalW to generate conserved protein domains not present in the Pfam and TIGRfam databases. *T. gondii*-specific domain alignments containing three or more proteins were considered true domains for the purpose of building families. Peptide sequences were extracted from alignments and searched back against the *T. gondii* proteome to look for additional members that may have been excluded during earlier stages due to the parameters used. Full-length protein sequences were then

grouped on the basis of the presence of Pfam/TIGRfam domains and potential novel domains, named "para" domains. Proteins with exactly the same combination of domains were classified into putative protein families. Gene families having two or more members organized as tandem arrays were identified using an in-house *perl* script.

The top five protein families containing novel para domains (TgFAMs A to E) were selected for further characterization. Briefly, for each family protein sequences were run through Phobius [22] a program that simultaneously identify the presence of potential signal peptides and transmembrane domains. De novo identification of conserved protein domains across members of the same gene family was carried out with MEME [23]. Expression levels for the TgFAMs were obtained from *T. gondii* Affymetrix Array data available from NCBI GEO records GSE32427 [15] and GSE51780. Data were normalized as described in [24].

Annotation of rRNA and tRNA genes

Sequences encoding for 5.8s, 18s and large rRNA subunits were extracted from GenBank entries L25635.1 and L37415.1, aligned to the new assembly with Nucmer [3] and automatically annotated with an in-house *perl* scripts. Transfer RNA encoding genes were predicted with tRNAscan-SE [25].

Annotation of other *T. gondii* reference strains

T. gondii ME49 genes were mapped at high and medium stringency to each *T. gondii* reference assembly with MUMmer and PASA respectively. Genes that mapped without errors with either method were promoted to working gene models while those that failed entered a second round of mapping with EVM. Briefly, coding sequences from failed genes were mapped to the target assembly with gmap [26]. Protein sequences from GenBank NR and Pfam seed databases and from the reannotated ME49 proteome were aligned to contigs with NAP and all RNAseq transcripts assembled with Trinity were mapped with PASA. Ab-initio gene predictions were performed with GlimmerHMM, Genezilla and two runs of Augustus, one of them using gmap-aligned genes as hints. Gene predictions, protein alignments and transcriptomic evidence were then integrated by EVM to annotate remaining working genes on the genome sequences. Lastly, annotated gene structures were updated with PASA based on aligned transcripts and manually inspected.

Functional annotation of protein-coding genes was performed following the same approach as with ME49 with the only exception that genes syntenic to ME49 annotations inherited their product names, Gene ontology (GO) terms and EC numbers while non-syntenic genes acquired their names

and other functional annotations from the output of JCVI's autonaming pipeline, PRIAM, ec2go and pfam2go mappings.

Sequencing and assembly of the *Hammondia hammondi* genome

Two 454 sequencing libraries (one fragment and one 3 kbp PE) and one Illumina 300 bp PE library were prepared from total gDNA extracted from *H. hammondi* oocysts (H.H.34 strain). The Illumina library was sequenced from both ends and 100 cycles in one eighth of an Illumina HiSeq 2000 lane while the fragment and 3 kbp PE 454 libraries were sequenced using two and one full 454 plates respectively. Sequencing reads from all libraries were screened for contamination and assembled with Newbler v2.6. The final *H. hammondi* assembly is 67.7 Mbp long with a contig N50 of 84,429 bp and an average sequencing depth of 66x (Supplementary Table 1).

Annotation of the *H. hammondi* assembly

Structural and functional annotations were carried out following a similar approach as the one described for other *T. gondii* reference genomes. In this case, however, GeneZilla, GlimmerHMM and Augustus were trained with a training set composed of 546 *H. hammondi* genes that were manually annotated and whose structures were both conserved in *T. gondii* and full-length supported by *T. gondii* transcripts. In addition, Augustus was run without hints or using evidence from *T. gondii* RNAseq/EST assembled transcripts or ME49 coding sequences.

Annotation of the apicoplast genomes from *T. gondii* reference strains and *H. hammondi*

Structural and functional annotation of the apicoplast rRNA and protein-coding genes were done manually using as reference the annotation of the apicoplast genome from the *T. gondii* RH strain (NC_001799.1). Prediction of apicoplast tRNA genes was performed with tRNAscan-SD.

Estimation of d_N/d_S ratios

Coding sequences from each cluster of orthologous genes from the 16 *T. gondii* reference strains were preprocessed using an in-house *perl* script in order to eliminate redundant sequences and potentially truncated or paralogous genes. Clusters with only one member left after the filtering step were discarded from the analysis. Afterwards, d_N/d_S ratios were calculated using a modified version of the Bioperl script *bp_pairwise_kaks.pl* (http://search.cpan.org/dist/BioPerl/scripts/utilities/bp_pairwise_kaks.pl). Briefly, this script aligns coding sequences in protein space with ClustalW [27] to ensure that codons are aligned properly, projects the alignments back into coding sequences and then estimates the non-synonymous versus synonymous substitution rates based on the maximum likelihood method of Yang [28] implemented in

the Codeml software from the PAML package [29]. For this analysis Codeml was run with the following parameters to estimate global d_N/d_S ratios for the entire multiple alignment: runmode = 0, seqtype = 1 and model = 0.

Analysis of orthologous genes using OrthoMCL

Annotated proteomes for *E. tenella* (strain Houghton, release date 2013-11-05), *S. neurona* (strain SN1, release date 2014-09-24), *N. caninum* (strain Liverpool, release date 2011-08-12), *T. gondii* (strain ME49, release date 2013-04-23), and *H. hammondi* (H.H.34 strain, release date 2014-09-03) were analyzed using OrthoMCL v2.0 [30,31] to define orthologous groups using the following parameters: Markov Clustering algorithm mcl ver 12.068 and default parameters (I=1.4, NCBI BLASTP ver 2.2.25 params = -a 1 -F 'm S' -v 100000 -b 100000 -Y 1300000 -e 1e-5).

Determination of Pfam domain abundance in coccidians

The proteomes from *T. gondii* ME49, *H. hammondi*, *N. caninum*, *S. neurona* and *E. tenella* were queried against the Pfam HMM database using HMMER3. Non-overlapping HMM hits above the trusted cutoff spanning at least 50% of the HMM and representing the best hit (lowest e-value) per protein region were selected for further quantification. For every genome, the abundance of each Pfam domain was then estimated as the number of protein sequences having at least one significant hit against a particular Pfam HMM.

Grouping GO annotations of orthologous groups

Clusters of orthologous groups present in the five coccidian genomes *E. tenella*, *S. neurona*, *N. caninum*, *T. gondii*, and *H. hammondi* were identified using OrthoMCL as defined above. These groups were functionally annotated using GO Slim terms, which are designed to group the many different GO terms into smaller groups of related processes (http://geneontology.org/page/go-slim-and-subset-guide). First, the list of GO terms associated with the proteins in the five organisms was inferred from their Pfam assignments, as described above. These terms were mapped onto the smaller-and-broader subset of generic GO Slim terms using map2slim script of the go-perl package, which maps a set of annotations up to their parent GO Slim terms. The GO Slim terms are mapped onto the OrthoMCL groups, and only groups containing the same annotation in >65% of its annotated members are considered.

Mapping non-synonymous SNPs and number of paralogs to iCS382 metabolic model of *T. gondii*

SNPs from the 16 *T. gondii* reference strains that correspond to the 382 proteins in the iCS382 metabolic pathway reconstruction of *T. gondii ME49* were downloaded from ToxoDB (http://www.toxodb.org/toxo-release4-0/home.jsp) [32]. SNPs were filtered using the following criteria: >80 % of the 16 strains contain an allele (major or minor) that is present in >80% of reads. Further, we considered only non-synonymous coding SNPs (missense and nonsense SNPs) that were present in at least 2 strains to negate the effects of random genetic drift. These criteria resulted in 2,697 nsSNPs. A normalized nsSNP score was generated for each EC number in iCS382 based on the following formula:

$$nsSNPs\ (normalized) = \frac{\sum_{j=1}^{Etot} \frac{number\ of\ nonsynonymous\ SNPs}{protein\ length}}{number\ of\ proteins\ with\ this\ EC\ in\ ME49\ (Etot)}$$

The number of paralogs for an EC was calculated for all 382 proteins from the orthologous groups of 16 reference *T. gondii* strains + *N. caninum* + *H. hammondi* generated using OrthoMCL at an inflation parameter of 1.4.

$$number\ of\ paralogs = \left(\frac{\sum_{j=1}^{Otot} number\ of\ proteins\ from\ 16\ Tg\ strains\ in\ the\ orthomcl\ group}{16}\right) - 1$$

Where *Otot* = number of orthoMCL groups containing a ME49 protein in iCS382 with this EC.

These values are mapped on to the iCS382 metabolic model and visualised using Cytoscape 3.1.1 (http://www.cytoscape.org/) [33].

SNP identification

Illumina reads for each of the 61 other genomes were aligned using Bowtie2 --end-to-end [34] against the ME49 reference genome assembly (release date 2013-04-23). Reads were realigned around gaps using the GATK toolkit [35] and a pileup file was generated using samtools [36]. VarScan [37] pileup2snp was used to make SNP calls with –min-coverage 5, -p-value 0.01 and –min-var-freq 0.8 (80% read consistency). These same parameters were utilized to make like-reference calls for each

strain at every position in the ME49 genome where any strain had a SNP call. This identified a total of 2,342,433 SNPs across all strains. Positions with informative base calls for all 62 strains were identified, generating a final list of 802,764 SNPs that were used for analysis.

Network and principal components analyses

Genome wide single nucleotide polymorphism (SNPs) were saved as FASTA files and directly incorporated into SplitsTree v4.4 [38] to generate unrooted phylogenetic networks using a neighbor-net method and 1,000 bootstrap replicates. Principal components analysis (PCA) was performed by eigenanalysis of a coancestry matrix implemented in fineSTRUCTURE, as described [39].

Chromosome Ia analysis

SNP data for ChrIa were plotted as a minimum spanning tree using SplitsTree v4.4 [38] with 2,000 spring embedded iterations. The 62 strains were clustered into four major groups denoted as monomorphic, divergent, 5'-chimeric, and 3'-chimeric chromosome Ia. SNPs present in each cluster were calculated using a custom script over a 10 kb moving window and plotted using Excel.

Admixture analysis

The population genetic structure of *T. gondii* was determined by an unsupervised clustering algorithm, ADMIXTURE [40] with ancestral clusters set from k = 1 through 10. The number of ancestral clusters k was determined by estimating the low cross-validation error (CV-error) for different k values using 5-fold CV.

Co-ancestry heatmap

We developed a co-ancestry heatmap by using the linkage model of ChromoPainter [41] and fineSTRUCTURE (http://www.paintmychromosomes.com) based on the genome-wide SNP data. For fineSTRUCTURE (version 0.02) [41], both the burn-in and Markov Chain Monte Carlo (MCMC) after the burn-in were run for 10,000 iterations with default settings. Each *T. gondii* strain was considered as a recipient of chunks of DNA donated by the other strains. Inference was performed twice at the same parameter values.

Estimating copy number variation (CNV)

Newly generated genomic sequence reads (454 and Illumina) for 62 strains of *T. gondii*, and previously generated sequences of the ME49 (ABPA00000000.2), GT1 (AAQM00000000.3), and VEG (AAYL00000000.2) strains were downloaded from NCBI (http://www.ncbi.nlm.nih.gov/). The programs sff-dump (NCBI SRA Toolkit) and sffToCA (Celera Assembler) were used to make fastq files from 454 based .sra files, and fastq-dump (NCBI SRA Toolkit) with the spilt-files option was used

to make fastq files from Illumina based .sra files. For each strain, the respective .sra files were used to align reads to the 14 ME49 reference chromosomes using Bowtie2 version 2.1.0 [42] with the end-to-end option. The read depth per bp, or read bases (RB), across 8,320 chromosomal-mapped genes was determined using samtools mpileup (SAMtools [36]). The mean of RB ($Gene n$) for base pairs spanning the genomic region of each gene was determined. The baseline 1X RB value for each strain was obtained by calculating the mean of RB ($1Xn$) and standard deviation of RB ($1Xs$) across all base pairs within genes (as one set) in the second and third quartile (4,160 genes) of the $Gene n$ distribution of the 8,320 protein coding genes. The CNV estimate was expressed as

$$CNVestimate = \frac{Gene n}{1Xm}$$

The cutoff for calling copy number variation (CNV) for a gene was based on

$$CNVcutoff = (CNVestimate >= 1Xm + (3 * 1Xs))$$

Plots were generated in R (http://www.r-project.org/). *T. gondii* gene families organized in tandem arrays were identified with an in-house *perl* script.

Analysis of OrthoMCL species-specific genes

Genes found to be specific to *T. gondii, H. hammondi, or N. caninum* based on OrthoMCL clustering were further analyzed using a combination of sequence alignment tools (**Supplemental Fig. 8A**). First, proteins encoded by species-specific genes were compared against the proteomes of the other two genomes with blastp using an e-value cutoff of $1x10^{-10}$ and at least 50% coverage of the shortest sequence. Genes that passed the cutoff were flagged as “Blastp hit” (see **Supplemental Fig. 8B** and **Supplemental Dataset 6**). Species-specific genes without significant blastp hits were then mapped in nucleotide space against the other two genomes with blastn (e-value ≤ $1x10^{-10}$ and coverage ≥ 70% of the query sequence) and those genes having significant and syntenic hits were selected for further analysis. A blastn hit was considered syntenic if the protein encoded by one of the genes located immediately upstream or downstream of the blastn hit on the subject genome was similar by blastp to the protein encoded by one of the genes at either side of the query sequence (e-value < $1x10^{-10}$ and coverage ≥ 50% of the shortest sequence). Species-specific genes without a significant blastn hit as described above were flagged as “Unique” genes (**Supplemental Fig. 8B**). Coding sequences (CDSs) from species-specific genes mapped by blastn were then aligned to the other two genomes using GMAP (with parameters -n 1 -A -a 1), a splice-aware nucleotide sequence alignment tool [43]. Proteins from species-specific genes whose CDSs did not aligned with GMAP were

then mapped to the other two genomes with tblastn (e-value ≤ $1x10^{-5}$). GMAP and tblastn hits were manually inspected to determine alignment coverage and the presence of non-sense mutations or frame-shifts in the subject sequence. Species-specific genes (or their protein sequences for tblastn) that mapped with either method with at least 70% coverage and did not present any in-frame STOP codon or frame-shift on the subject sequence were labeled as "Full Length" (**Supplemental Fig. 8B**). Genes (or their proteins) that mapped with less than 70% coverage or presented an in-frame STOP codon or frame-shift on the subject sequence were flagged as "Alternative Gene Model" and constitute potential pseudogenes or functional genes with an altered gene structure compared to the query sequence (**Supplemental Fig. 8B**). Remaining unmapped genes were added to the gene pool flagged as "Unique".

RNA-seq analysis of unique and alternative gene models in *T. gondii*.

Assembled *T. gondii* RNA-seq transcripts generated during the annotation phase of the project were mapped using blastn to CDSs from *T. gondii* genes that were classified as Unique, Alternative Gene Models, BlastP Hits and Full Length based on their comparison to *H. hammondia* and *N. caninum*. Those CDSs that aligned to a transcript across their entire length with at least 95% identity were flagged as supported by RNAseq data. The statistical significance of the difference in relative abundance of each gene category with or without RNAseq support was assessed using the Fisher's exact test with the R function *fisher.test*.

Estimation of maximum alternative allele frequency scores and minimum sequencing depth.

Genome sequencing reads used to assemble the genomes of *H. hammondi* (32,612,714 Illumina reads) and *T. gondii* ME49 strain (4,697,063 454 and Sanger reads) were mapped to their respective genomes with Bowtie2 followed by SNP and INDEL identification with the utilities *mpileup* from *samtools* and *call* from *bcftools* with parameters "-cv -p 1" to ensure that all alternative alleles were reported, independently of their allele frequency. Thereafter, the maximum alternative allele frequency (MAAF) score, defined as the maximum allele frequency reached among the collection of alternative alleles identified in every genomic region of interest, was calculated with an in-house *perl* script. To estimate the minimum sequencing depth (MSD) reached by each locus hit by alternative gene models we calculated the sequencing depth at every nucleotide position of that region with the utility *depth* of the program *samtools* using default parameters and then MSD was calculated using an in-house *perl* script. Binning of MAAF scores and MSD data was carried out with the R program *hist*.

Regions of co-inheritance

To determine the extent of recombination and co-inheritance of blocks between strains, we examined all possible pair-wise comparisons between the 62 strains (62 x 62). There are 3,844 pair-wise comparisons of which 1,953 are unique. The number of SNPs per 10 kb window across the 14 chromosomes for each pair-wise strain comparison was determined. Low SNP regions (regions of recent co-inheritance, or shared blocks) were identified for 10 kb windows that have 5 or fewer SNPs across a continuous stretch of 10 windows (100 kb) allowing for the intermittent outlier. The ratio of windows meeting this criterion out of all 10 kb windows (6,202) was used as the percentage of the genome two strains share as recently co-inherited (referred to as % shared blocks). A heatmap was generated using the R function heatmap.2 (gplots library (http://www.r-project.org/)) with hierarchical clustering on the % shared blocks value. To analyze the composition of genes within shared regions, strains were grouped by Clade based on this hierarchical clustering: Clade A - 18 strains, Clade B - 8 strains, Clade C - 9 strains, Clade D - 8 strains, Clade F - 9 strains. Clade E was not included in this analysis as the strains within this Clade are highly similar. The number of SNPs per 10kb window were averaged for all strains within a Clade, and chromosomal regions with low SNP density were identified as above using 10 kb windows that had 3 or fewer SNPs across a continuous stretch of 10 windows (100 kb), allowing for intermittent outliers.

Identification of SPD genes and clustering within the genome

We identified genes that belong to the SPD families (i.e. *MIC*, *GRA*, *ROP*, *SRS* and *TgFAM*) based on the annotation of ME49 accounting for CNV in determining the gene number. We then mapped the position of the SPDs onto the assembled ME49 genome and defined those that fell into conserved or non-conserved regions. To determine if gene type was independent of region type we compared the observed frequency of SPDs and non-SPD genes in conserved vs. non-conserved regions of the genome using a Chi-squared analysis. The null hypothesis was that the distribution would be random, and there would be no difference between observed and expected. A P value of ≤ 0.05 was considered significant cause for rejection of the null hypothesis.

Ancestry of conserved and non-conserved regions

The regions of the genome that are "conserved" were defined as the union of low SNP regions for Clades A, B, C , D, and F. We ignored Clade E because it is highly clonal. From these positions, we separated the SNP matrix (all SNPs for 62 strains) into those that were conserved vs. divergent (non-conserved) for the analyses. We reconstructed phylogenetic trees for the conserved and non-

conserved regions using maximum likelihood as implemented in RAxML version 7.3.0 with the GTR+GAMMA model [44]. We calculated the standardized Robinson-Foulds (RF) distance [45] between the conserved and non-conserved trees. The standardized RF distance equals the proportion of negative branches in the two trees. In addition, we generated 500 bootstrap trees for the non-conserved region, and calculated the standardized RF distances between the 500 bootstrap trees and the ML tree for the non-conserved region. Those 500 distances represent the variation of the tree estimate due to mutation when the true tree is the non-conserved tree. We used the maximum distance as the threshold to identify the conserved tree that is significantly different from the non-conserved tree. We also estimated phylogenetic tress for the sequence blocks in conserved and non-conserved regions of Clades C, D, and F (where the null hypothesis for random distribution had been rejected). In each of the conserved and non-conserved regions, genes were separated into SPD and non-SPD genes and separate trees were generated for each using 100 bootstrap replicates using RAxML with the GTR+Gamma model described above. The 100 bootstrap trees were combined into a consensus with support values. Trees were considered congruent if they had no conflicting branches with bootstrap support of > 95%.

Phylogeny

To generate a phylogeny that spans across the Apicomplexa, we chose an ortholog shared by all the major taxa that is defined by OrthoMCL OG5_0126701. The gene id for *T. gondii* is TGME49_249810 and it encodes a 2,749 amino acid protein. In different apicomplexans, this gene is annotated as a DEAD box helicase or activating signal cointegration 1 complex subunit 3. The corresponding protein has regions of high conservation allowing broad phylogenetic comparisons, as well as variable domains that provide better resolution within closely related lineages. Phylogenetic trees were constructed using the Neighbor Joining algorithm with 1,000 Bootstrap replicates as implemented in Geneious ver. 7.1.5 (http://www.geneious.com, [46]) and visualized with FigTree ver. 1.4.0 (http://tree.bio.ed.ac.uk/software/figtree/).

Synteny

Briefly, the OrthoMCL ortholog clusters (see above) were reformatted to represent each pair found in the cluster outside of self-matches. Syntenic blocks were generated between all combinations of genomes as described in [47]. A minimum number of three genes in a 25 kb search window up and downstream from each orthologous gene was required to form a syntenic block. Intervening non-

syntenic genes were allowed. Custom scripts were used to calculate the total number of syntenic blocks between genomes as well as the number and percentage of genes present in syntentic blocks. The MCSCAN [48] output was formatted for appropriate input to Circos ver. 0.51 [49] for visualization.

Chromosome painting

Local admixture analyses using an enhanced ADMIXTURE algorithm [40] were conducted on blocks of size 1,000 SNPs on each of the 14 chromosomes of *T. gondii.* Local admixture was used to simultaneously optimize the number of ancestral states for a given genomic region and assign each of the 62 strains to clusters representing these ancestral states. Specifically, for each block of SNPs, we performed local admixture analysis with the number of ancestral states k = 2-7, and then chose the one with the minimum cross-validation (CV) error as the optimal number of ancestral states. The ".Q" output file for the optimal ancestral states provided the probability of each strain being assigned to each ancestral state. If the probability was greater than 0.9, the strain was assigned to the corresponding ancestral state. For each sequence block present in an ancestral state cluster, we colored the region according to clades represented in Figure 4a based on majority rule (*i.e*. we counted how many sequences in the ancestral state cluster belonged to 1 or more of the 6 groups in Figure 4a and we assigned the color that represented the largest number of sequences to all of the sequences in that ancestral state cluster).

References

1 Su, C. L. *et al.* Globally diverse *Toxoplasma gondii* isolates comprise six major clades originating from a small number of distinct ancestral lineages. *Proc. Natl. Acad. Sci. (USA)* **109**, 5844-5849(2012).

2 Myers, E. W. *et al.* A whole-genome assembly of Drosophila. *Science* **287**, 2196-2204(2000).

3 Delcher, A. L., Phillippy, A., Carlton, J. & Salzberg, S. L. Fast algorithms for large-scale genome alignment and comparison. *Nucleic Acids Res* **30**, 2478-2483(2002).

4 Miller, J. R., Koren, S. & Sutton, G. Assembly algorithms for next-generation sequencing data. *Genomics* **95**, 315-327(2010).

5 Sonnhammer, E. L., Eddy, S. R. & Durbin, R. Pfam: a comprehensive database of protein domain families based on seed alignments. *Proteins* **28**, 405-420(1997).

6 Huang, X., Adams, M. D., Zhou, H. & Kerlavage, A. R. A tool for analyzing and annotating genomic sequences. *Genomics* **46**, 37-45(1997).

7 Reid, A. J. *et al.* Comparative genomics of the apicomplexan parasites *Toxoplasma gondii* and *Neospora caninum*: Coccidia differing in host range and transmission strategy. *PLoS Pathog* **8**, e1002567(2012).

8 Grabherr, M. G. *et al.* Full-length transcriptome assembly from RNA-Seq data without a reference genome. *Nat Biotechnol* **29**, 644-652(2011).

9 Haas, B. J. *et al.* Improving the Arabidopsis genome annotation using maximal transcript alignment assemblies. *Nucleic Acids Res* **31**, 5654-5666(2003).

10 Majoros, W. H., Pertea, M. & Salzberg, S. L. TigrScan and GlimmerHMM: two open source ab initio eukaryotic gene-finders. *Bioinformatics* **20**, 2878-2879(2004).

11 Stanke, M., Tzvetkova, A. & Morgenstern, B. AUGUSTUS at EGASP: using EST, protein and genomic alignments for improved gene prediction in the human genome. *Genome Biol* **7 Suppl 1**, S11 11-18(2006).

12 Birney, E., Clamp, M. & Durbin, R. GeneWise and Genomewise. *Genome Res* **14**, 988-995(2004).

13 Blanco, E., Parra, G. & Guigo, R. Using geneid to identify genes. *Curr Protoc Bioinformatics* **Chapter 4**, Unit 4 3(2007).

14 Haas, B. J. *et al.* Automated eukaryotic gene structure annotation using EVidenceModeler and the Program to Assemble Spliced Alignments. *Genome Biol* **9**, R7(2008).

15 Fritz, H. M. *et al.* Transcriptomic analysis of toxoplasma development reveals many novel functions and structures specific to sporozoites and oocysts. *PLoS One* **7**, e29998(2012).

16 Haft, D. H., Selengut, J. D. & White, O. The TIGRFAMs database of protein families. *Nucleic Acids Res* **31**, 371-373(2003).

17 Marchler-Bauer, A. *et al.* CDD: conserved domains and protein three-dimensional structure. *Nucleic Acids Res* **41**, D348-352(2013).

18 Emanuelsson, O., Brunak, S., von Heijne, G. & Nielsen, H. Locating proteins in the cell using TargetP, SignalP and related tools. *Nat Protoc* **2**, 953-971(2007).

19 Claudel-Renard, C., Chevalet, C., Faraut, T. & Kahn, D. Enzyme-specific profiles for genome annotation: PRIAM. *Nucleic Acids Res* **31**, 6633-6639(2003).

20 Eddy, S. R. Accelerated Profile HMM Searches. *PLoS Comput Biol* **7**, e1002195(2011).

21 Jaccard, P. The Distribution of the Flora in the Alpine Zone. *The New Phytologist*, 37-50(1912).

22 Kall, L., Krogh, A. & Sonnhammer, E. L. A combined transmembrane topology and signal peptide prediction method. *J Mol Biol* **338**, 1027-1036(2004).

23 Bailey, T. L. *et al.* MEME SUITE: tools for motif discovery and searching. *Nucleic Acids Res* **37**, W202-208(2009).

24 Behnke, M. S., Zhang, T. P., Dubey, J. P. & Sibley, L. D. Toxoplasma gondii merozoite gene expression analysis with comparison to the life cycle discloses a unique expression state during enteric development. *BMC Genomics* **15**, 350(2014).

25 Lowe, T. M. & Eddy, S. R. tRNAscan-SE: a program for improved detection of transfer RNA genes in genomic sequence. *Nucleic Acids Res* **25**, 955-964(1997).

26 Wu, T. D. & Watanabe, C. K. GMAP: a genomic mapping and alignment program for mRNA and EST sequences. *Bioinformatics* **21**, 1859-1875(2005).

27 Thompson, J. D., Gibson, T. J. & Higgins, D. G. Multiple sequence alignment using ClustalW and ClustalX. *Curr Protoc Bioinformatics* **Chapter 2**, Unit 2 3(2002).

28 Goldman, N. & Yang, Z. A codon-based model of nucleotide substitution for protein-coding DNA sequences. *Mol Biol Evol* **11**, 725-736(1994).

29 Yang, Z. PAML: a program package for phylogenetic analysis by maximum likelihood. *Comput Appl Biosci* **13**, 555-556(1997).

30 Fischer, S. *et al.* Using OrthoMCL to assign proteins to OrthoMCL-DB groups or to cluster proteomes into new ortholog groups. *Curr Protoc Bioinformatics* **Chapter 6**, Unit 6 12 11-19(2011).

31 Li, L., Stoeckert, C. J., Jr. & Roos, D. S. OrthoMCL: identification of ortholog groups for eukaryotic genomes. *Genome Res* **13**, 2178-2189(2003).

32 Song, C. *et al.* Metabolic reconstruction identifies strain-specific regulation of virulence in *Toxoplasma gondii*. *Mol Syst Biol* **9**, 708(2013).
33 Shannon, P. *et al.* Cytoscape: a software environment for integrated models of biomolecular interaction networks. *Genome Res* **13**, 2498-2504(2003).
34 Langmead, B. & Salzberg, S. L. Fast gapped-read alignment with Bowtie 2. *Nat Methods* **9**, 357-359(2012).
35 DePristo, M. A. *et al.* A framework for variation discovery and genotyping using next-generation DNA sequencing data. *Nat Genet* **43**, 491-498(2011).
36 Li, H. *et al.* The Sequence Alignment/Map format and SAMtools. *Bioinformatics* **25**, 2078-2079(2009).
37 Koboldt, D. C. *et al.* VarScan: variant detection in massively parallel sequencing of individual and pooled samples. *Bioinformatics* **25**, 2283-2285(2009).
38 Huson, D. H. & Bryant, D. Application of phylogenetic networks in evolutionary studies. *Mol. Biol. Evol.* **23**, 254-267(2006).
39 Price, A. L. *et al.* Principal components analysis corrects for stratification in genome-wide association studies. *Nat Genet* **38**, 904-909(2006).
40 Alexander, D. H., Novembre, J. & Lange, K. Fast model-based estimation of ancestry in unrelated individuals. *Gen. Res.* **19**, 1655-1664(2009).
41 Lawson, D. J., Hellenthal, G., Myers, S. & Falush, D. Inference of population structure using dense haplotype data. *PLoS Genet* **8**, e1002453(2012).
42 Langmead, B., Trapnell, C., Pop, M. & Salzberg, S. L. Ultrafast and memory-efficient alignment of short DNA sequences to the human genome. *Genome Biol* **10**, R25(2009).
43 Wu, T. D. & Wantanabe, C. K. GMAP: a genomic mapping and alingment program for mRNA and EST sequences. *Bioinformatics*, 1859-1875(2005).
44 Stamatakis, A. RAxML-VI-HPC: maximum likelihood-based phylogenetic analyses with thousands of taxa and mixed models. *Bioinformatics* **22**, 2688-2690(2006).
45 Robinson, D. R. & Foulds, L. R. Comparison of phylogenetic trees. *Mathmatical Biosciences* **53**, 131-147(1981).
46 Kearse, M. *et al.* Geneious Basic: an integrated and extendable desktop software platform for the organization and analysis of sequence data. *Bioinformatics* **28**, 1647-1649(2012).
47 DeBarry, J. D. & Kissinger, J. C. Jumbled genomes: missing Apicomplexan synteny. *Mol Biol Evol* **28**, 2855-2871(2011).
48 Tang, H. *et al.* Unraveling ancient hexaploidy through multiply-aligned angiosperm gene maps. *Genome Res* **18**, 1944-1954(2008).
49 Krzywinski, M. *et al.* Circos: an information aesthetic for comparative genomics. *Genome Res* **19**, 1639-1645(2009).
